# *hsp70 *genes in the human genome: Conservation and differentiation patterns predict a wide array of overlapping and specialized functions

**DOI:** 10.1186/1471-2148-8-19

**Published:** 2008-01-23

**Authors:** Luciano Brocchieri, Everly Conway de Macario, Alberto JL Macario

**Affiliations:** 1 University of Florida, Department of Molecular Genetics and Microbiology and UF Genetics Institute, Gainesville, FL 32610, USA; 2 Center of Marine Biotechnology, University of Maryland Biotechnology Institute, Baltimore, MD 21202, USA

## Abstract

**Background:**

Hsp70 chaperones are required for key cellular processes and response to environmental changes and survival but they have not been fully characterized yet. The human *hsp70*-gene family has an unknown number of members (eleven counted over ten years ago); some have been described but the information is incomplete and inconsistent. A coherent body of knowledge encompassing all family components that would facilitate their study individually and as a group is lacking. Nowadays, the study of chaperone genes benefits from the availability of genome sequences and a new protocol, chaperonomics, which we applied to elucidate the human *hsp70 *family.

**Results:**

We identified 47 hsp70 sequences, 17 genes and 30 pseudogenes. The genes distributed into seven evolutionarily distinct groups with distinguishable subgroups according to phylogenetic and other data, such as exon-intron and protein features. The N-terminal ATP-binding domain (ABD) was conserved at least partially in the majority of the proteins but the C-terminal substrate-binding domain (SBD) was not. Nine proteins were typical Hsp70s (65–80 kDa) with ABD and SBD, two were lighter lacking partly or totally the SBD, and six were heavier (>80 kDa) with divergent C-terminal domains. We also analyzed exon-intron features, transcriptional variants and protein structure and isoforms, and modality and patterns of expression in various tissues and developmental stages. Evolutionary analyses, including human *hsp70 *genes and pseudogenes, and other eukaryotic *hsp70 *genes, showed that six human genes encoding cytosolic Hsp70s and 27 pseudogenes originated from retro-transposition of HSPA8, a gene highly expressed in most tissues and developmental stages.

**Conclusion:**

The human *hsp70*-gene family is characterized by a remarkable evolutionary diversity that mainly resulted from multiple duplications and retrotranspositions of a highly expressed gene, HSPA8. Human Hsp70 proteins are clustered into seven evolutionary Groups, with divergent C-terminal domains likely defining their distinctive functions. These functions may also be further defined by the observed differences in the N-terminal domain.

## Background

Molecular chaperones encompass several families, play important physiological roles, some of which essential for life, and are key components of anti-stress mechanisms in all organisms, tissues and cells [1]. The Hsp70 family is thought to be required for various cellular processes and for the response to environmental changes and survival [2,3]. Although there is no doubt about the biological and medical importance to the human Hsp70 chaperone family, a coherent, unifying informational framework encompassing all members, that would facilitate their understanding individually and as a group is lacking. A framework of multiple data pertaining to all family members is necessary for planning future experiments to further elucidate the evolution, synthesis and maturation, structure, functions, trafficking and pathology, of these genes and proteins. Likewise, this framework would provide an essential context for interpreting the results and for inferring new properties that can be submitted to experimental testing.

Hsp70 proteins and their parent genes have been studied in prokaryotic and eukaryotic organisms chiefly because of their participation in protein folding under normal and stress conditions, and of their potential role in ageing and pathology, e.g., cancer, and apoptosis [4-7]. Many *hsp70 *genes have been reported, generating a confusing array of data, variants, and nomenclatures in databases and printed literature. The use of different names for the same *hsp70 *gene and/or protein and the use of the same name for various *hsp70 *genes and/or proteins make interpretation of the reported results very difficult if not impossible. Furthermore, in many instances it is not entirely clear if reference is made to the gene or its protein product, and in other instances, it is impossible to know exactly what gene or protein in the family is being considered, since only the word Hsp70 is used without further clarification.

Analyses of *hsp70 *genes at the genome level have been carried out for a few organisms, such as *Caenorhabditis *[8], *Drosophila *[9,10], and *Ciona intestinalis *[11]. An earlier attempt, more than ten years ago, at defining the human Hsp70 family was based on printed literature searches and yielded 11 genes [12]. Since then, no systematic, comprehensive analysis of human *hsp70 *genes has to our knowledge been published.

In this report, we provide a comprehensive characterization of the extended family of human *hsp70 *genes and their proteins based on genomic, structural, and evolutionary analyses. The overall picture is that of a diversified family of proteins that achieved diversity at various levels: genetic, transcriptional, and post-transcriptional. The implications of this diversity for function, cell and tissue localization, and pathology can be predicted to be wide and important for life and to have a significant impact on biomedical sciences. The unified and coherent matrix of data we generated pertaining to evolution, genetics, transcription, structure, anatomic distribution, and regulation of the entire human Hsp70 family should provide a solid context for future studies and for the development of therapeutic means specifically targeted to well-defined family members [13].

## Results

### Human *hsp70 *genes

We carried out extensive literature and database searches and found 130 human Hsp70 proteins with corresponding names and identifiers. Similarity analysis of these proteins revealed 86 groups of non-identical Hsp70 sequences, which in turn could be clustered by further analysis into 13 groups. From each of these 13 groups, we chose a representative sequence to query the human genome NCBI Build 36.1 for conserved and diverged sequences, as described in Methods, and we found 47 loci encoding *hsp70*-like sequences. Of these sequences, 16 corresponded to genes recognized in the genome annotation. Another sequence was identified as an *hsp70 *gene, named HSPA7, which is transcribed under certain conditions although it is considered by some as a pseudogene (see later). The main characteristics of the 17 *hsp70 *genes are shown in Table 1. We identified thirty other sequences as belonging to the *hsp70*-gene family, but they had features such as frame-shifts and in-frame stop codons indicating that they are pseudogenes. The main characteristics of these 30 *hsp70*-related pseudogenes are shown in Table 2. The chromosomal distribution of all *hsp70 *genes and pseudogenes is displayed in Table 3. The genes and pseudogenes were distributed over 18 chromosomes, some of which had both protein-coding genes and pseudogenes, while chromosomes 15–17, 19 and 22 had neither. Hsp70 genes and pseudogenes were distributed among chromosomes irrespective of their evolutionary relations, with the exception of the triad of genes HSPA1A, HSPA1B, HSPA1L, all encoded in close proximity on chromosome 6, and of the pair of genes HSPA6, HSPA7, encoded near each other on chromosome 1.

**Table 1:** *hsp70* genes in the human genome: Main characteristics (NCBI Build 36.1)^a^

Name/ID	Location	S	Start/End	nt	aa	Ex	Is
Hsp70 kDa 6 (HSP70B')/HSPA6	1q23.3	+	159,761,073/159,763,001	1,929	643	1	1
Hsp70 kDa 7 (HSP70B)/HSPA7	1q23.3	+	159,842,705/159,844,628^b^	1,924	641	1	1
Hsp70 kDa 4-like/HSPA4L	4q28.1	+	128,923,156/128,973,476	50,321	839	19	1
Hsp70 kDa 9B/HSPA9B	5q31.2	-	137,919,628/137,938,906	19,279	679	17	1
Hsp70 kDa 4/HSPA4	5q31.1	+	132,415,842/132,468,024	52,183	840	19	a
			132,415,842/132,468,024	52,183	148	5	b
Hsp70 kDa 1-like/HSPA1L (HSP70-Hom)	6p21.33	-	31,885,806/31,887,728	1,923	641	1	1
Hsp70 kDa 1A/HSPA1A (HSP70-1)	6p21.33	+	31,891,513/31,893,435	1,923	641	1	1
Hsp70 kDa 1B/HSPA1B (HSP70-2)	6p21.32	+	31,903,707/31,905,629	1,923	641	1	1
Hsp70 kDa 5 (Grp78)/HSPA5	9q33.3	-	127,038,695/127,043,226	4532	654	8	1
Hsp70 kDa 12A/HSPA12A	10q25.3	-	118,424,285/118,456,787	32,503	675	12	1
Hsp70 kDa 14/HSPA14	10p13	+	14,920,408/14,953,608	33,201	509	14	1
			14,920,408/14,924,181	3,774	88	4	2
Hsp70 kDa 8/HSPA8	11q24.1	-	122,433,655/122,437,242	3,588	646	8	1
			122,433,655/122,437,242	3,588	493	7	2
150 kDa oxygen-regulated protein/HYOU1	11q23.3	-	118,421,518/118,432,082	10,565	999	25	1
			118,421,518/118,432,082	10,565	999	25	1
			118,421,518/118,431,751	10,234	964	24	2
			118,424,940/118,432,082	7,143	687	16	3
Hsp105 kDa/HSPH1	13q12.3	-	30,609,458/30,633,719	24,262	858	18	α
			30,609,458/30,633,719	24,262	814	17	β
Hsp70 kDa 2/HSPA2	14q23.3	+	64,077,321/64,079,237	1,917	639	1	1
Hsp70 kDa 12B/HSPA12B	20p13	+	3,667,322/3,680,810	13,489	686	12	1
Stress 70 protein chaperone/STCH	21q11.2	-	14,667,812/14,677,311	9,500	471	5	1

^a^Abbreviations and symbols: ID, identifying designation used in this work; S, DNA strand; Start/End, chromosome positions comprising the coding region (see Table S1, Additional File 1 for start and end positions of the primary transcript); nt, number of nucleotides between Start and End; aa, length in amino acid of the protein product(s); Ex, exons (non coding exons excluded); Is, isoform names (for the 150 kDa oxygen-regulated protein/HYOU1 there are four mRNA variants but only three protein isoforms because two variants have the same translation initiation site).

^b^Bypassing an internal frame-shift (see text). The putative coding region would otherwise end at position 159,843,805 and encode for a protein 367 aa long.

**Table 2:** *hsp70*-related pseudogenes in the human genome: Main characteristics (NCBI Build 36.1)

Name	**Chr^a^**	S	Start/End	nt	aa	F	Stops	I
P1.1	1	+	38,947,258/38,949,148	1,891	628	4	0	-
P1.2	1	+	245,459,865/245,461,803	1,939	649	4	1	-
P2.1	2	-	209,645,569/209,647,064	1,496	498	1	7	-
P2.2	2	+	222,534,878/222,536,560	1,683	562	3	0	-
P2.3	2	-	235,317,093/235,318,989	1,897	534	7	25	(2)
P3.1	3	+	139,081,827/139,083,766	1,940	641	4	4	-
P3.2	3	-	19,956,765/19,959,824	3,060	846	12	7	2(3)
P4	4	+	189,871,681/189,873,662	1,982	661	10	15	-
P5	5	+	129,503,623/129,505,555	1,933	646	7	9	-
F5	5	-	63,644,128/63,644,314	187	63	1	2	-
F6.1	6	-	151,776,456/151,776,854	399	131	5	1	-
F6.2	6	+	147,708,650/147,708,925	276	92	0	0	-
F6.3	6	+	47,769,360/147,769,647	288	97	2	0	-
F6.4	6	-	80,187,101/80,187,848	748	219	1	10	(1)
P7.1	7	+	10,457,465/10,459,400	1,936	646	1	2	-
P7.2	7	+	84,495,080/84,496,823	1,744	589	10	9	-
F7	7	+	97,272,880/97,273,101	222	74	0	1	-
P8	8	+	30,214,440/30,218,052	3,613	612	6	4	2
F8.1	8	+	47,581,377/47,581,649	273	91	0	3	-
F8.2	8	-	47,778,468/47,778,785	318	106	0	4	-
P9	9	+	30,977,881/30,980,601	2,721	538	8	14	1
P10	10	+	27,043,495/27,044,962	1,468	435	6	18	(2)
P12.1	12	+	4,076,878/4,078,947	2,070	649	5	3	(1)
P12.2	12	+	110,304,007/110,307,864	3,858	653	4	5	5
F13	13	-	88,996,627/88,997,451	825	238	3	10	(1)
F18.1	18	-	25,090,760/25,090,992	233	78	1	0	-
F18.2	18	-	62,025,362/62,025,705	344	78	1	2	(1)
PX.1	X	-	120,164,760/120,166,561	1,802	602	3	3	-
PX.2	X	-	123,166,030/123,167,337	1,308	336	5	7	1
PX.3	X	-	113,915,646/113,917,590	1,945	668	2	10	(1)

^a^Abbreviations and symbols: Chr, chromosome; S, DNA strand; Start/End, positions at which the homology region begins and ends, respectively; nt, number of nucleotides between start and end, i.e., length of the recognized homology region; aa, number of amino acids, namely codons in the predicted pseudocoding region; F, frame shifts; Stops, in-frame stop codons; I, apparent pseudointrons. In parentheses signifies that the inserted element is short.

**Table 3:** Human chromosomes with *hsp70 *genes and *hsp70*-related pseudogenes

Chr^a^	Protein-coding gene	Pseudogene
1	HSPA6, HSPA7	P1.1, P1.2
2		P2.1, P2.2, P2.3
3		P3.1, P3.2
4	HSPA4L	P4
5	HSPA9B, HSPA4	P5, F5
6	HSPA1L, HSPA1A, HSPA1B	F6.1, F6.2, F6.3, F6.4
7		P7.1, P7.2, F7
8		P8, 8.1, F8.2
9	HSPA5	P9
10	HSPA12A, HSPA14	P10
11	HSPA8, HYOU1	
12		P12.1, P12.2
13	HSPH1	F13
14	HSPA2	
18		F18.1, F18.2
20	HSPA12B	
21	STCH	
X		PX.1, PX.2, PX.3

^a^Chr, chromosome

### Variants and isoforms

The protein isoforms shown in Table 1 pertain in the majority of cases to known mRNA variants. In the case of HSPA7, a transcription product was terminated by a stop codon after 367 amino acids. However, a complete conserved Hsp70 protein of 641 amino acids could be encoded bypassing a frame shift at codon position 340. For the HYOU1 gene, we found four mRNA variants two of which using the same translation initiation site, corresponding to three protein isoforms (Table 1).

Besides the described mRNA variants indicated in Table 1, many more processed variants for the majority of the genes were predicted from the analysis of EST (Expressed Sequence Tag) and SAGE (Serial Analysis of Gene Expression) data. Prediction of transcript variants based on these data (ECgene database [14,15] and NCBI AceView database, D. Thierry-Mieg, J. Thierry-Mieg, M. Potdevin and M. Sienkiewicz, unpublished) are displayed and compared in Table 4, which also shows that ECgene and AceView predictions differ considerably. For example, for HSPA8 at least 19 protein variants are predicted by ECgene vs. 14 predicted by AceView, 13 vs. 10 variants are predicted for HSPA1A or HSPA1B, 3 vs. 15 are predicted for HYOU1, 1 vs. 12 are predicted for HSPA6, and similar differences were observed for most of the other genes. These inconsistencies could result from the use of somewhat different datasets, or from different quality-control procedures applied to the EST data. In any case, EST analysis suggested that there is a significantly greater number of transcript variants and isoforms for *hsp70 *genes than previously suspected.

**Table 4:** Predicted mRNA variants of human *hsp70* genes^a^

Gene	ECgene data^b^		ECgene variant predictions^b^		AceView variant predictions^c^	
	
	mRNAs	ESTs	Transcripts	Proteins	Transcripts	Proteins
HSPA8	9 (11)	3156 (4729)	21 (95)	19 (52)	14	14
HSPA12A	2 (2)	108 (112)	4 (6)	4 (6)	4	4
HSPA12B	1 (1)	37 (38)	1 (2)	1 (2)	2	2
HSPA9B	5 (5)	906 (1010)	10 (267)	3 (42)	9	9
HSPA4	5 (5)	441 (464)	12 (30)	5 (11)	8	8
HSPA4L	n.a.^d^	n.a.	n.a.	n.a.	1	1
HSPH1	8 (8)	405 (444)	9 (55)	7 (25)	11	11
HYOU1	4 (4)	469 (482)	3 (13)	3 (7)	17	15
HSPA14	6 (6)	209 (226)	10 (17)	5 (9)	8	8
STCH	3 (3)	209 (210)	3 (5)	2 (3)	2	2
HSPA2	3 (5)	187 (261)	6 (7)	6 (7)	1	2
HSPA1A/B	8 (8)	1411 (1411)	21 (21)	13 (13)	1	10
HSPA1L	2 (2)	36 (36)	2 (2)	1 (1)	2	4
HSPA6	2 (4)	2 (2)	1 (2)	1 (1)	15	12
HSPA7	n.a.	n.a.	n.a.	n.a.	n.a.	n.a
HSPA5	5 (6)	1174 (1305)	10 (18)	6 (10)	4	4

^a^Within parentheses are the total number of predicted transcripts and proteins including those of lower confidence level.

^b^Data from [14,15].

^c^NCBI AceView Database [41].

^d^Not available.

### Evolutionary history of the human *hsp70 *genes

**Figure 1:**
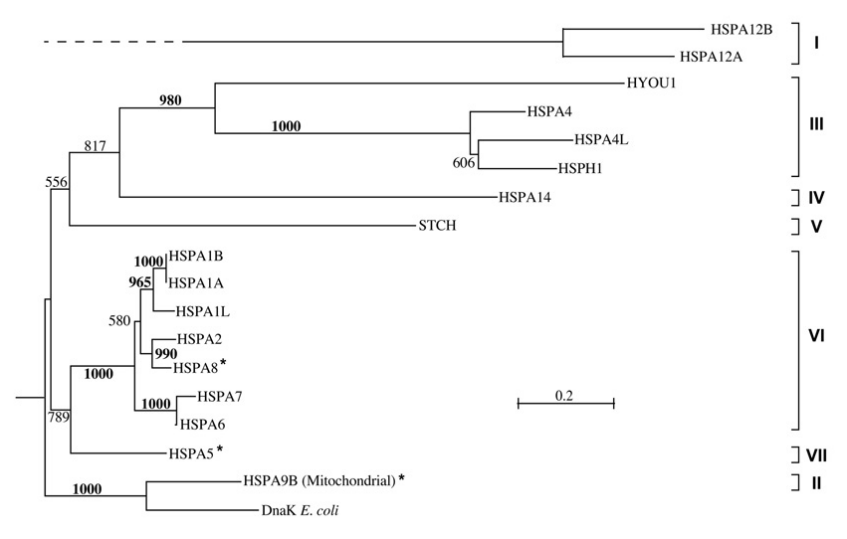
**Phylogenetic tree of the 17 human Hsp70 proteins encoded in the genes studied in this work**. The seven distinct evolutionary groups supported by bootstrap values (1000 samples) equal or greater than 85% are indicated by brackets to the right. Alignments were obtained with Clustal W [42] and the evolutionary tree was obtained using the neighbor-joining algorithm [38] with the distance transformation method of Kimura [39], as implemented in Clustal W. A similar tree (not shown) was obtained using the maximum likelihood approach implemented in the program PHYML [40].

We computed phylogenetic trees of the human *hsp70 *genes based on the alignment of their protein products using both distance and maximum-likelihood procedures (see Methods) obtaining with both methods highly similar results. We excluded from the overall alignment the sequences of HSPA12A and HSPA12B, which would have significantly reduced the useful size of the alignment. A phylogenetic tree of the proteins encoded by the 17 *hsp70 *genes (Figure 1) distinguished seven major evolutionarily related groups, which we defined by a bootstrap-support values over 85%. These Groups are indicated in Figure 1 with brackets. Group I was composed of the most diverged sequences, HSPA12A and HSPA12B, of uncertain relation with other eukaryotic and prokaryotic Hsp70s. It had previously been noticed that HSPA12A and HSPA12B branch in an intermediate position between eukaryotic and prokaryotic *hsp70 *sequences [16] but the origin of these two genes was not investigated further. Although conservation of a few sequence motifs clearly identifies these two genes as members of the extended *hsp70 *gene family, their sequence conservation was insufficient to allow for a reliable, accurate determination of their evolutionary position compared to other human Hsp70s.

Group II was composed of the mitochondrial protein HSPA9B, considered of alphaproteobacterial origin [17,18], which, accordingly, clustered with the DnaK sequence from *E. coli *(Gammaproteobacteria) with very high bootstrap support.

Group III encompassed the 105/110 kDa proteins HSPA4, HSPA4L, HSPH1 (clustered with 100% bootstrap support) and the more distantly related sequence HYOU1, coding for the 170 kDa protein Grp170. HSPA14 was also related to proteins in Group III but with lower (81.7%) bootstrap support than that (85% or higher) we adopted to identify closely related sequences as members of a distinct Group. Therefore, we classified HSPA14 separately in Group IV. Also joined to the sequences of Groups III and IV, was the sequence STCH, but with considerably lower bootstrap support (55.6%) than that required to differentiate the Groups, and thus we assigned STCH to another group, i.e., Group V.

Group VI was composed of sequences clustered with 100% bootstrap support and, among them, we distinguish three subgroups, one including HSPA1A, HSPA1B and HSPA1L, a second including HSPA8 and HSPA2, and a third including HSPA6 and HSPA7.

Group VII included sequence HSPA5, expressed in the endoplasmic-reticulum (ER), which was joined to Group VI with lower bootstrap support (78.9%) than the minimum adopted for distinguishing the main groups.

We considered the sequences from Groups II (mitochondrial), VI (cytosolic/nuclear) and VII (ER resident) typical Hsp70 proteins because they had conserved ATP-binding and substrate-binding domains (ABD and SBD, respectively; see later). In contrast, we considered the sequences in all the other Groups atypical because they had in what would be the SBD of the typical Hsp70 molecules no equivalent of this domain, or incomplete or highly diverged equivalents.

### Origin and evolution of the human *hsp70*-related pseudogenes

**Figure 2:**
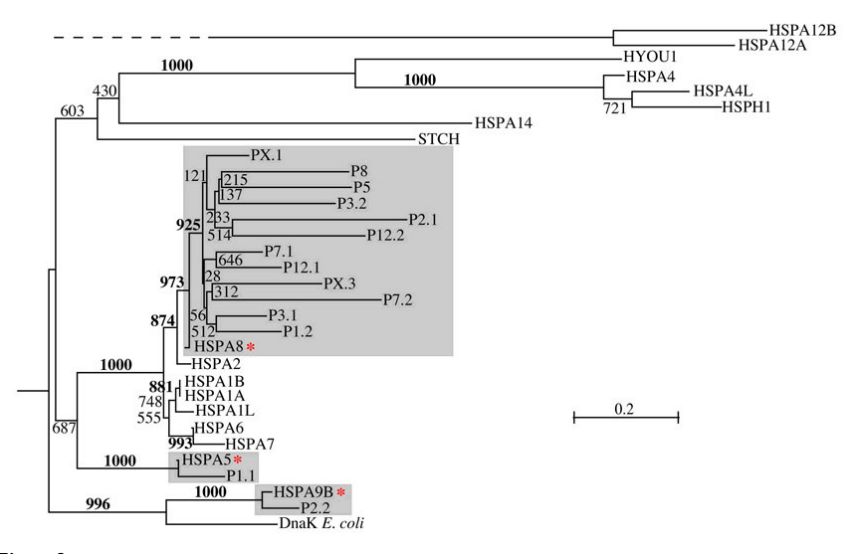
**Phylogenetic tree of the 17 human Hsp70 proteins and the proteins that would be encoded in the related pseudogenes identified in this work**. Same as tree shown in Figure 1 but including the most conserved pseudogenes (see text). Shaded areas highlight each group of pseudogenes with the gene from which they originated, indicated by an asterisk. A similar tree (not shown) was obtained using the maximum-likelihood approach implemented in the program PHYML [40]. See legend for Figure 1 and text for details on Methods.

An evolutionary tree of the proteins that would be encoded by the pseudogenes identified in this work in relation to the proteins encoded in the 17 *hsp70 *genes is shown in Figure 2. We included in the tree the 16 less corrupted sequences out of the 30 pseudogenes. The evolutionary tree indicates that the vast majority of the *hsp70 *pseudogenes originated from HSPA8. We also identified the Hsp70 sequence most similar to each of the 30 pseudogenes. The results are shown in Table 5 also in comparison to sequence characterizations for the pseudogenes available at the Pseudogene Database [19]. Twenty-three of the 30 pseudogenes were predicted in the Pseudogene Database, while seven pseudogenes were not and are newly reported here.

**Table 5:** *hsp70*-related pseudogenes in the human genome: Type and evolutionary origin

Name^a^	Type PD	Ori PD	Ori	Sim	Name	Type PD	Ori PD	Ori	Sim
P1.1	P	HSPA5	HSPA5^1^	90	P7.2	P	HSPA8	HSPA8^1^	71
P1.2	P	-	HSPA8^1^	88	F7	-	-	HSPA8^2^	46
P2.1	P	HSPA8	HSPA8^1^	68	P8	D	-	HSPA8^1^	76
P2.2	P	-	HSPA9B^1^	83	F8.1	A	-	HSPA8^2^	46
P2.3	A	-	HSPA8^2^	27	F8.2	-	-	HSPA8^2^	33
P3.1	P	-	HSPA8^1^	87	P9	D	-	HSPA8^2^	31
P3.2	A	HSPA6	HSPA8^1^	74	P10	-	-	HSPA8^2^	23
P4	D	-	HSPA8^2^	30	P12.1	D	HSPA8	HSPA8^1^	88
P5	P	HSPA8	HSPA8^1^	73	P12.2	D	HSPA8	HSPA8^1^	69
F5	-	-	HSPA8^2^	70	F13	A	-	HSPA8^2^	30
F6.1	D	-	HSPA8^2^	36	F18.1	A	-	HSPA9B^2^	87
F6.2	-	-	HSPA8^2^	27	F18.2	-	-	HSPA8^2^	51
F6.3	A	-	HSPA8^2^	87	PX.1	P	-	HSPA8^1^	79
F6.4	-	-	HSPA8^2^	30	PX.2	D	HSPA6	HSPA8^2^	73
P7.1	P	HSPA8	HSPA8^1^	90	PX.3	P	HSPA8	HSPA8^1^	83

^a^Abbreviations and symbols: pseudogenes with a predicted pseudocoding region ≥ 300 aa are called P, and those shorter than 300 aa are called F (fragment); PD, Pseudogene Database [19]; Type PD, predicted pseudogene type as per the Pseudogene Database; Ori PD, pseudogene origin as per the Pseudogene Database; Ori, pseudogene origin determined in this work, based on phylogenetic-tree branching (1 superscript) and based on similarity with the closest sequence (2 superscript); Sim, percent similarity (identities plus conservative substitutions of the predicted pseudoprotein); P, processed (retrotranscribed); A, ambiguous; D, duplicated.

Phylogenetic analysis of the most conserved pseudogenes and detailed comparison of the most corrupted ones with the closest sequence among the 17 *hsp70 *genes, revealed that 27 of the 30 pseudogenes originated from the HSPA8, two from HSPA9B, and one from HSPA5 (Figure 2 and Table 5).

### Exon/intron structure of the human *hsp70 *genes

The number of exons for each gene are shown in Table 1, and a detailed characterization of the genome position and length of each exon is shown in Table S1, Additional File 1. An analysis of the exon/intron structure of these genes provided important insights into their evolutionary relations. With the exception of all but one of the genes in group VI (see below), all *hsp70 *genes are multiexonic, ranging from a minimum of five exons in STCH up to 25 exons in HYOU1. Details on the intron-exon structure of individual gene sequences and their relations within and between the *hsp70 *Groups are provided below.

#### Group I

The two similar sequences HSPA12A and HSPA12B possessed an identical exon-intron structure with 12 exons within the coding region (Table S1, Additional File 1). HSPA12B also possessed an intron in its 5' UTR region. The HSPA12A and HSPA12B proteins were quite dissimilar from the other Hsp70 sequences and, within the few regions of homology we found no splice site in common with sequences from other groups.

#### Groups II, VI and VII (Figure S1, Additional File 1)

Sequences from groups II, VI, and VII included typical Hsp70 sequences expressed, respectively, in the mitochondrion, cytosol/nucleus, and ER. The genes HSPA1A, HSPA1B and HSPA1L (frequently called *Hsp70-1*, *Hsp70-2 *and *Hsp70-Hom*, respectively) from Group VI are positioned within the MHC-III region on the short arm of chromosome 6 [7,20], and are intronless. In fact, among the genes in Group VI, only HSPA8 possessed multiple exons (8 and 7 in the two isoforms) within the coding region. We compared the splice positions in HSPA8 (Group VI), HSPA5 (Group VII) and HSPA9B (Group II), and found a splice site conserved within one codon position in HSPA8 (between codons 188 and 189) and HSPA5 (between codons 164 and 165), and we also found one splice site conserved within two codon positions between HSPA8-2 (within codon 463) and HSPA9B (between codons 505 and 506).

#### Group III

Within Group III (Figure S2, Additional File 1), the closely related HSPA4, HSPA4L and HSPH1 sequences conserved a common exon-intron structure of 18–19 exons, further supporting the cohesiveness of this group. Among these sequences, HSPH1 was distinguished by fusion of the exons labeled VII and VIII of HSPA4 and HSPA4L into a single exon named VII (Table S1, Additional File 1), with an apparent intron-loss/intron-insertion event in the subgroup. A remarkable feature of the exon-intron structure of this subgroup was that most splice sites and all of the last seven splice sites (all but two of the splice sites within the substrate-binding domain) were between codons (i.e., the splice sites did not disrupt codons). Within Group III, HYOU1 was the most distantly related. It included 27 splice sites, of which 25 (24 or 16 in alternatively spliced variants) were within the coding region. Ten of these sites were within the ABD (8–9 splice sites were within this region in the other sequences of Group III). One splice site was conserved throughout all sequences in Group III (the third splice site between codon positions 102 and 103 of the HSPA4, HSPH1 subgroup and between codon positions 88 and 89 of HYOU1-1). Six other splice sites were either in close proximity (within 5 codons) or bordering diverged inserted elements. Besides the ABD region, HYOU1 shared similarity with other sequences of Group III in a short segment immediately following the ABD and mostly within a region of about 200 codons between positions 710 and 917. This relatively conserved region included five splice sites in HYOU1 as well as in HSPA4, HSPA4L, and HSPH1. One of these five splice sites was conserved among all sequences (between codons 604 and 605 in HSPA4L, and between nucleotides 2 and 3 of codon 722 in HYOU1). In conclusion, by comparing HYOU1 splice sites with those of the other members of Group III, we recognized two conserved splice sites (one in the ABD and one in the SBD) in this Group. We also found a few other splice sites in close proximity within conserved sequence elements or in correspondence to inserted/deleted elements.

We compared Group III with other sequences (Figure S3, Additional File 1) and showed a splice site in homologous positions in HSPA4, HSPA4L, and HSPH1 (positioned between nucleotides 1 and 2 of codon 329 in HSPA4L) and in the mitochondrial gene HSPA9B (positioned between codons 394 and 395). Also, we found a splice site between codons 379 and 380 in HSPA4L within two homologous codon positions (within position 374) of a corresponding splice site in HSPA8 isoform 1.

#### Groups IV and V

Group IV (Figures S2 and S3, Additional File 1) was composed of one sequence, HSPA14, which in the phylogenetic tree shown in Figure 1 was associated to Group III with bootstrap support 81%, marginally below the threshold of bootstrap support (85%) that we chose to define evolutionary Groups. Within the coding regions of HSPA14-a there were 14 splice sites (in HSPA14-b there were 4 splice sites within the coding region and one in the 3' UTR). In HSPA14-a, we found a splice site within codon 191 in a position homologous to a splice site in HYOU1 (in which, however, the splice site was between codons 226 and 227). A second splice site of HSPA14-a, within codon 297, was homologous to a corresponding splice site in HSPA4L (within codon 303) and closely related sequences. A third splice site, positioned after codon 90 of HSPA14-a, coincided with a splice site in a homologous position in HSPA5 (after codon 118) and in STCH (after codon 122), although weak conservation in this region made the alignment less reliable. A splice site of HSPA14-a at codon 191 was within a short variable region in which splice sites occurred also in HYOU1 (between codons 226 and 227 of isoforms 1 and 2) and in HSPA9B (within codon 239). Finally, a splice site within codon 245 (between nucleotides 2 and 3) of HSPA14-a was within one homologous codon of another splice site in HSPA9B (between nucleotides 2 and 3 of codon 293).

The *STCH *(Group V) gene had 5 splice sites in its coding region. Since STCH was weakly associated (with 55.6% bootstrap support) in the phylogenetic tree to the sequences of Group III and to HSPA14 (Group IV), it was interesting to see whether some similarities in the exon-intron patterns of these sequences could be detected but we did not detect any common splice site associating STCH (Group V) to Group III. However, we did find a splice site in common among STCH (between codons 122 and 123), HSPA14-a (between codons 90 and 91), and HSPA5 (between codon 118 and 119).

### Evolution of eukaryotic Hsp70 sequences

We reconstructed the phylogenetic tree of eukaryotic typical Hsp70s, including the human ones (Groups II, VI and VII), and 137 other typical sequences from completely sequenced eukaryotic genomes. The tree is shown in Figure S4, Additional File 1, in which all branches with bootstrap support < 40% have been collapsed into multi-partitions. The tree constructed using all the information available, did not have sufficient resolution to discern most of the ancient phylogenetic relations. However, the tree did provide several important pieces of information. For example, thirty-three eukaryotic mitochondrial sequences clustered with the *E. coli *DnaK in a separate group, which roots the tree of other eukaryotic sequences. Apart from a few protist sequences of uncertain origin and a sequence from Microsporidia (Fungi), the cluster of ER proteins, including Protists, Plants, Fungi, and Animals (including human HSPA5) separated early, as in the trees of human sequences (e.g., Figure 1), indicating that the separation of ER and cytosolic sequences happened early in the evolution of eukaryotes. The cytosolic sequences presented several lineages of uncertain relations (Figure S4, Additional File 1), many involving sequences from Protists, two from Fungi and a third set from Plants. A major cluster of sequences with weak bootstrap support (47.3%) included all human cytosolic sequences. Within this cluster, two subclusters with relatively high support (77.7% and 72.0%) included, respectively, the human HSPA8 and HSPA2. A third cluster with lower support (51.8%) included HSPA1A, HSPA1B and HSPA1L.

**Figure 3:**
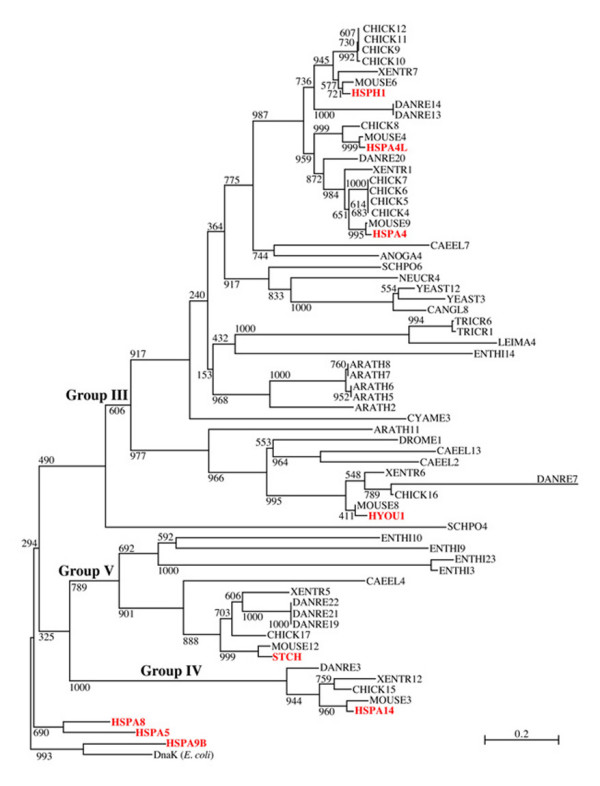
**Phylogenetic tree of atypical eukaryotic *hsp70 *genes**. Included are the proteins encoded in the atypical human *hsp70 *genes (Groups III, IV, and V in Figure 1) and in the *hsp70 *genes of eighteen other completely sequenced eukaryotic genomes. Sequences from the latter eighteen non-human genomes were identified using as queries human proteins from Groups II, VI, and VII and the SSPA procedure [36]. The tree is rooted by the DnaK sequence (AAC73125.1) from *E. coli *(DnaK *E. coli*) and by representatives of typical human Hsp70s (from Groups II, VI, and VII). See legend to Figure 1 and text for details on methods and calculation of bootstrap values. Human proteins are in red. Acronyms in black indicate the eukaryotic genomes in which other *hsp70 *genes were found, as follows: ANOGA: *Anopheles gambiae *(Insects); ARATH: *Arabidopsis thaliana *(Plants); CANGL: *Candida glabrata *(Fungi); CAEEL: *Caenorhabditis elegans *(Nematoda); CHICK: *Gallus gallus *(Birds); CYAME: *Cyanidioschyzon merolae *(Red alga); DANRE: *Danio rerio *(Fish); DROME: *Drosophila melanogaster *(Insects); ENTHI: *Entamoeba histolytica *(Protists); LEIMA: *Leishmania major *(Protists); MOUSE: *Mus musculus *(Mammals); NEUCR: *Neurospora crassa *(Fungi); SCHPO: *Schizosaccharomyces pombe *(Fungi); TRYCR: *Trypanosoma cruzi *(Protists); XENTR: *Xenopus tropicalis *(Amphibians); YEAST: *Saccharomyces caerevisiae *(Fungi).

Figure 3 displays an evolutionary tree of eukaryotic atypical Hsp70 proteins from completely sequenced genomes, including representatives of Protists, Plants, Fungi, and Animals encompassing Nematodes, Insects, Fish, Amphibians, and Mammals. In these genomes, we found sequences most closely related to human Group III, Group IV, and Group V. The tree is rooted by a group encompassing DnaK from *E. coli *and human mitochondrial HSPA9B and include, for comparison, other representatives of the human typical Hsp70s such as the ER-residing HSPA5 and the cytosolic HSPA8 proteins. As mentioned earlier, we excluded from the tree sequences of Group I (i.e., HSPA12A and B), which were too diverged from the other sequences to be used in the alignment. The tree in Figure 3 shows, similarly to those in preceding figures, that the relation between Groups III, IV, and V are uncertain, a likely consequence of their ancient divergence. Also noteworthy is that contrary to the trees in the previous figures, in the tree shown in Figure 3 Groups IV and V (represented in humans by HSPA14 and STCH, respectively) appear to have diverged from a common ancestor, with weak bootstrap support (32.5%) but consistent nonetheless with their similarity in molecular weight (about 60 kDa) and sequence domain composition (see later). Interestingly, the cluster including STCH (Group V) contained sequences from animals such as *C. elegans *(but not *Drosophila*) and from Protists (*Enthamoeba*) but not from Plants or Fungi, whereas the cluster containing HSPA14 (Group IV) included only sequences from Vertebrates. Apart from a single sequence (SCHPO4) from *S. pombe *of uncertain classification, the sequences of the high molecular weight proteins, corresponding to the human Group III, were distinguished into the two subgroups of 105/110 kDa and 170 kDa as in the human tree shown in Figure 1, with high bootstrap support (91.7% and 97.7%, respectively). Sequences of the 105/110 kDa subcluster (including the human HSPH1, HSPA4L and HSPA4) were present in various Protists and in higher eukaryotes, suggesting that they originated from an ancient gene duplication event. A duplication of the gene into two branches, corresponding respectively to the human sequences HSPA4 and HSPH1 (representing two of the three subgroups of Group III; Figure 1) appears to have occurred early in the evolution of the Vertebrate lineage, before the appearance of Tetrapods (amphibians, reptiles, birds, and mammals). A subsequent duplication of HSPA4 into two separate genes (corresponding to HSPA4 and HSPA4L) seems also to have occurred before the appearance of Tetrapods (as indicated by the position in the tree of the sequence DANRE20). Interestingly, no representatives of HSPA4L from fish or amphibian were found. Multiple paralogs from the HSPH1 and HSPA4 gene-lines seem to have been recently generated in the chicken genome. Unexpectedly, the cluster containing the 170 kDa subgroup of the human Group III (HYOU1) included only sequences from animals and plants, suggesting gene loss or divergence in other lineages, including Fungi.

**Figure 4:**
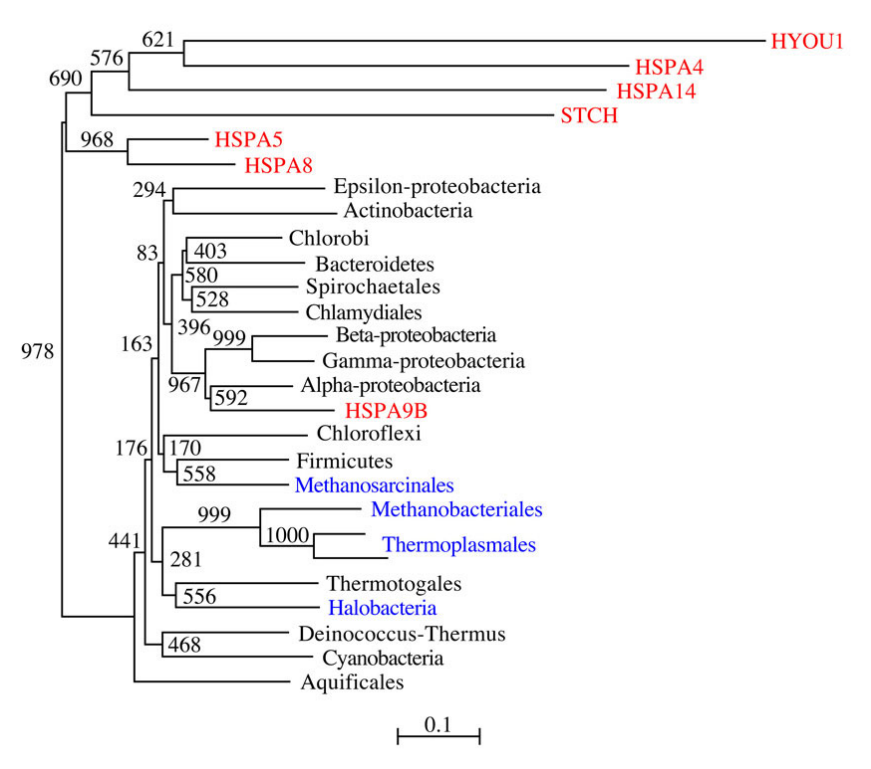
**Phylogenetic tree of human Hsp70 proteins and prokaryotic DnaK**. Included are all human Hsp70 proteins except the highly diverged HSPA12A and HSPA12B. One sequence was chosen for each prokaryotic (bacterial in black, and archaeal in blue) group. Human proteins are in red.

We also studied the evolutionary relations of all human Hsp70 proteins (excluding HSPA12A and HSPA12B) with prokaryotic DnaK(Hsp70) proteins, resulting in the evolutionary tree displayed in Figure 4 that shows the close relation of HSPA9B (mitochondrial) with alpha-proteobacterial sequences. The tree also suggests that all other human *hsp70 *genes originated and differentiated from a unique prokaryotic ancestor of uncertain identity.

### Evolution of HSPA12

**Figure 5:**
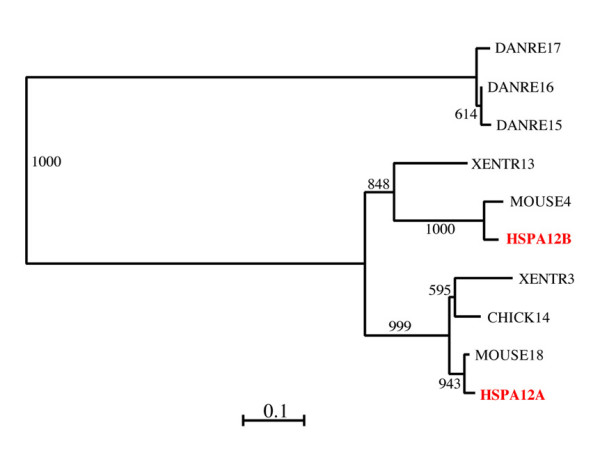
**Phylogenetic tree of eukaryotic Hsp70 protein 12**. The tree includes the human HSPA12A and HSPA12B proteins and the homologs found in eighteen sequenced eukaryotic genomes. See legend for Figure 3 and text for methods and other details. Homologs of human HSPA12A and HSPA12B were found only in vertebrates. The evolutionary tree suggests that the human genes originated from a unique progenitor also appearing in fish possibly due to a duplication event that occurred in Tetrapoda before the divergence of Amphibia. Only one HSPA12 copy was found in reptiles and birds, suggesting gene loss (or extreme divergence) of one HSPA12 copy from this lineage.

We also searched for the genes encoding proteins like HSPA12A and HSPA12B in the sequenced genomes examined in Figure 3. We found homologs of these genes only in fish (*Danio*), Amphibian (*Xenopus*), Bird (chicken), and Mammal (human and mouse), a finding that together with their evolutionary relations shown in Figure 5, suggests that the various *HSPA12 *genes evolved at an early stage in the evolution of vertebrates (or that versions of the gene that diverged earlier do not conserve significant similarity to current human homologs). We found in *Danio rerio *(fish) three recently diverged copies of the gene (similarities 97–99%) that were quite diverged from the human and amphibian sequences (similarities in the range 25–32%). Chicken had only one copy, which was most similar to the human HSPA12B (92% similarity), whereas we found two copies of this gene in mammals and amphibians. The evolutionary tree of the gene suggests that the amphibian and mammalian copies resulted from a unique duplication event that occurred in the tetrapod lineage before the radiation of the amphibian and mammalian lineages. Within each of the one amphibian and two mammals examined, the two gene copies had similarities in the range 61–65%. Each paralog was quite conserved among the three groups, with similarities between mammals and amphibian of 82–83% for the HSPA12A paralog, and of 71–73% for the HSPA12B paralog, whereas between human and mouse similarities were 97% and 94%, respectively. The phylogenetic relations of the vertebrate lineages imply that one of the two copies of the gene has been lost from the chicken genome. Interestingly, we identified in the chicken genome a non-annotated sequence of 186 nt (positions 45,897,943–45,898,128 of chromosome 1) in the complementary strand, encoding a protein with 56.5% identity to a segment of the annotated chicken *hspa12 *gene. This short gene is possibly a remnant of *HSPA12A*.

### Hsp70 proteins: Structural domain characteristics of the groups and subgroups

**Figure 6:**
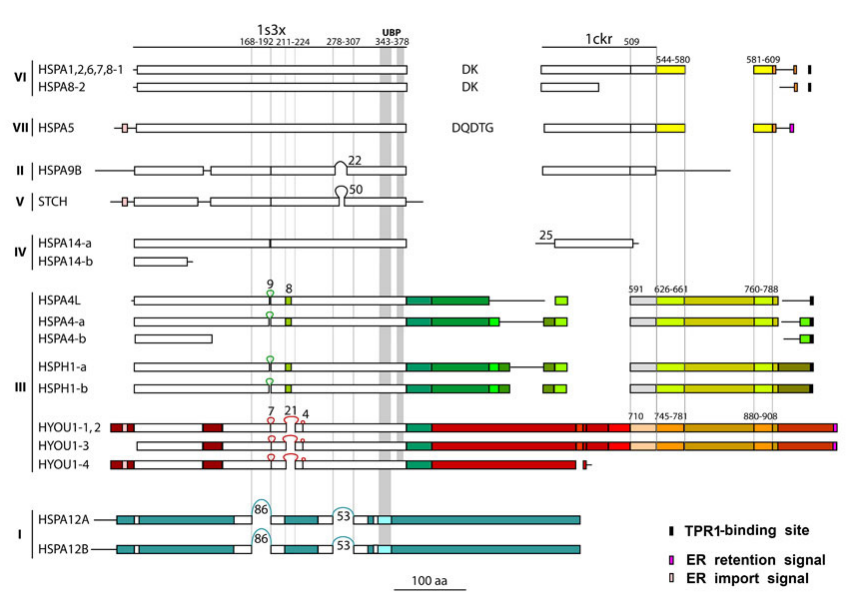
**Structural features of human Hsp70 proteins**. Typical Hsp70 structures comprise two major domains: ATP-binding domain (ABD) and substrate-binding domain (SBD). The regions comprising the crystal structure of the ABD (1s3x [21], human HSPA1A, positions 1–382) and the NMR structure of the SBD (1ckr [22], rat HSPA8, corresponding to human HSPA8 and HSPA1A homologous positions 385–543) are shown as lines at the top of the figure. Domains conserved in human Hsp70 sequences within these regions are shown as aligned white boxes. Other domains conserved among groups of sequences are shown as aligned boxes of matching colors. Roman numerals to the left indicate the Hsp70 evolutionary groups. UBP, ubiquitin-binding peptide; TPR1, tetratrico peptide repeat 1; ER, endoplasmic reticulum. See text for other details.

Conserved and unique sequence elements of the different members of the human Hsp70 protein family were identified and are schematically displayed in Figure 6. Hsp70 proteins typically comprise two major domains: an ATP-binding domain (ABD) and a substrate-binding domain (SBD). Most of the ABD of the human HSPA1A (positions 1–382) has been resolved by crystallography [21], PDB identifier 1s3x, and most of the SBD of the rat HSPA8 (corresponding to human HSPA8 and homologous to human HSPA1A positions 385–543) has been elucidated by NMR [22], PDB identifier 1ckr. The positions of the sequences corresponding to the solved structures of the ABD and SBD domains are shown in Figure 6 aligned to the corresponding sequence domains conserved among the human Hsp70 proteins, drawn as white boxes. Other domains conserved among groups or subgroups of sequences are shown as aligned boxes of matching colors.

The ABD was conserved in most proteins; segments of it were present in all of them, thus providing one parameter for recognizing them as members of the *hsp70 *family. We found variants of ABD in the two subgroups of Group III distinguished by high bootstrap support, namely the HSPA4-HSPA4L-HSPH1 subgroup, and the HYOU1 subgroup, with regions of differentiation and inserted elements (shown in different color and as loops with length indicated, respectively) in correspondence to exposed loops in the crystal structure of HSPA1A (see below). In HSPA12A and HSPA12B (Group I), most of the ABD was highly divergent, with scattered conserved regions that allowed their identification as members of the extended Hsp70 protein family. An incomplete ABD was present in HSPA14-b (Group IV), and in HSPA4-b (Group III).

The SBD was considerably less conserved than the ABD. For example, the typical SBD as observed in HSPA8 was entirely conserved only in the sequences of Groups II, VI, and VII. A C-terminal portion of proteins in Groups VI and VII of about 70 amino acids, comprising the 35 C-terminal residues included in the crystal structure and 66 residues not included in the crystal structure, shown in yellow in Figure 6, was found in the proteins of Group III (HSPA4L, HSPA4-a, and HSPH1-a and -b, light green segments; and HYOU1-1, 2, and 3, orange segments). In all of these proteins, this sequence segment was interrupted by a long insertion characteristic of Group III, somewhat differentiated in the HSPA4L and HSPA4-a sequences in which it is shown in greenish yellow, compared to HYOU1 in which it is shown in brownish. In contrast, we did not find in the proteins of Group I any sequence similarity to the SBD of Group VI sequences.

Other noteworthy findings are listed below (see Figure 6):

1) HSPA8-2 and HSPA14-a had only a partial, N-terminal sequence of the SBD;

2) The HSPA4 and HSPH1 subgroups were characterized by distinctive domains (dark green) different from the ABD and SBD. HYOU1 had inserted long, unique elements (reddish) that separated shorter regions of homology to the SBD of Group VI;

3) The sequence C-terminal to the part of SBD resolved in the rat HSPA8 structure (1ckr) was not conserved in HSPA9B, in which it was instead more similar to the corresponding prokaryotic sequence of DnaK (shown by a straight line);

4) HSPA12A and HSPA12B (Group I) had, as mentioned earlier, portions (white segments) of the canonical ABD typical of the other groups (markers of the Hsp70 family), but did not share any similarity with their SBD;

5) The ABD of STCH was characterized by a long insertion of 50 aa (positions 311–360) that substituted residues 285–292 of HSPA1A, corresponding to a loop connecting two beta-strands in the apical part of one of the two ATP-binding jaws of the ABD. This inserted element was not predicted to have an ordered secondary structure (as determined by the secondary-structure prediction program PHD). An unrelated inserted element in the same position was present in HSPA9B (22 aa at positions 323–344, substituting 18 aa at positions 278–295 in HSPA1A). A more extended region of divergence occurred in the same region also in HSPA12A and B, in which positions 278–307 (30 aa) of HSPA1A were substituted by a diverged element of 53 aa (positions 402–454 in HSPA12B). These changes may reflect functional differentiation endowing the HSPA12 proteins with specific capabilities not shared with other Hsp70s.

6) Another major region of differentiation corresponded to positions 211–224 of HSPA1A (1s3x), which were substituted by a diverged segment of 21 aa in HYOU1 (positions 245–265 in HYOU1 isoforms 1 and 2). A shorter overlapping peptide within the same region (corresponding to positions 210–217 in 1s3x) was diverged in other Group III sequences (shown in green and corresponding to positions 215–222 in HSPA4 and positions 213–220 in HSPA4L and HSPH1). An insertion of four aa (sequence LRLR) was present in HYOU1 (positions 277–280 in isoforms 1–2) inserted in correspondence to positions 234 and 235 of HSPA1A. These positions are part of an alpha-helix whose structure most likely influences the ATP-binding site's architecture and thereby its functionality.

7) Still another region that was modified in all atypical Hsp70s (typical proteins are those in Groups II, VI, and VII, as mentioned earlier) corresponded to positions 189–192 of HSPA1A (1s3x), in which this short segment of four aa was substituted by longer loops (nine aa) in the HSPA4 and HSPH1 subgroups and in the HYOU1 subgroup (seven aa insertion). The same region represents the C-terminal part of a longer segment of 25 aa (positions 168–192) substituted by a long inserted element of 86 aa in HSPA12A and HSPA12B (positions 229–314 of HSPA12B). While positions 189–192 formed an exposed loop in HSPA1A, the longer peptide of 25 aa involved more substantial structural elements, including an extended loop followed by a mostly-buried alpha-helix.

8) The interaction of Hsp70 proteins with Hsp90 proteins is mediated by the adaptor protein Hop, whose TPR1 domain specifically recognizes an Hsp70 C-terminal TPR1 domain-binding site [23]. This site was conserved in the sequences of Group VI, in HSPA4L, in HSPA4 (both isoforms), and in HSPH1 (both isoforms), ending with the acidic-aliphatic-acidic triad EVD, EVD, DID and DLD, respectively. This finding suggests that the functions of the Hsp70s just mentioned include interacting with Hsp90. In contrast, we did not find the TPR1 domain-binding site in HSPA5, HSPA9B, STCH, HSPA14, HYOU1, HSPA12A, or HSPA12B.

9) In STCH, a sequence segment in close proximity to the C-terminus of the ABD (shown as UBP for ubiquitin-binding peptide in Figure 6) was shown to be involved in interactions with the ubiquitin-system proteins Chap1 and Chap2 [24]. This peptide corresponds to positions 411–445 of the STCH sequence. Its homologous positions 343–378 in the HSPA1A structure [21] correspond to an exposed extended loop (425-DPNTSVDPD-433) anchored by two surrounding alpha-helices. The UBP (with alpha-helical regions shaded in Figure 6) was conserved in all human Hsp70s with the exceptions of HSPA12A and B, in which only the N-terminal alpha-helix sequence was somewhat recognizable. This conservation was particularly evident in the typical Hsp70s (Groups II, VI, and VII), in which the UBPs had similarities of 54–64% with the STCH counterpart, suggesting that functions associated with the ubiquitin proteasome system (and hence with protein-degradation pathways) are shared by the majority of Hsp70 proteins, including atypical ones.

10) Consistent with their localization in the ER, we identified ER-import signal peptides in HSPA5, STCH and HYOU1 isoforms 1, 2 and 4 (i.e., excluding isoform 3). An ER-retention signal was identified in HSPA5 and in HYOU1 isoforms 1, 2, and 3, but not in the microsome-associated protein STCH.

### Patterns of *hsp70 *gene expression in tissues, developmental stages, and cellular compartments

The expression levels of *hsp70 *genes in various tissues can be estimated by assessing the relative number of ESTs per tissue type. Summary results on the frequency of human *hsp70 *gene ESTs per tissue type obtained from the NCBI UniGene database [25] are displayed in Table 6. Noteworthy were the relatively very high expression levels of HSPA8 and HSPA1A in vascular tissue and in the spleen, respectively. At the opposite end of the spectrum, the expression levels of HSPA1L and HSPA12B were very low, or undetectable in the majority of tissues examined. All genes were expressed each in only one tissue at much higher levels than in the other tissues, with one exception, HSPA12A, which was preferentially expressed in two tissues, parathyroid and nerve. These findings suggest that accurate determination of gene regulation and protein function for different *hsp70 *paralogs should be experimentally investigated for each pertinent tissue and that findings pertaining to a tissue must not be assumed to apply to other tissues.

**Table 6:** Expression levels of *hsp70 *genes in various human tissues^a^

TISSUE/GENE	HSPA9B	HSPA5	HSPA1A	HSPA1B	HSPA1L	HSPA8	HSPA2	HSPA6	STCH	HSPA14	HSPA4	HSPA4L	HSPH1	HYOU1	HSPA12A	HSPA12B
**Blood**	336	172	546	72	0	2412	9	118	36	36	18	0	109	54	0	0
**Bone**	341	505	150	0	0	1326	0	41	54	54	164	13	123	109	0	13
**Bone marrow**	448	529	61	81	0	1691	40	20	40	40	61	0	142	448	0	0
**Brain**	364	99	805	235	3	3355	753	14	127	58	45	43	308	120	88	6
**Connective**	289	373	476	56	18	2943	37	18	84	37	74	0	93	130	37	9
**Adipose**	73	591	2885	591	**73**	1035	0	73	0	73	73	0	147	73	73	0
**Ear**	177	118	769	0	0	591	118	0	0	0	59	59	177	0	59	0
**Liver**	306	316	397	62	0	1198	4	0	23	28	52	19	47	306	0	0
**Pancreas**	150	388	480	155	0	818	4	18	27	36	82	4	77	128	9	0
**Adrenal gland**	538	348	1805	411	0	3959	63	95	95	31	158	0	190	221	95	0
**Parathyroid**	616	47	0	0	0	568	0	0	0	**331**	0	47	47	0	**237**	0
**Thyroid**	523	**1571**	776	271	0	1318	0	0	0	72	36	18	144	198	162	18
**Pituitary gland**	223	503	167	0	55	1734	55	55	0	55	167	55	**559**	55	223	0
**Placenta**	219	293	169	13	0	856	219	53	79	66	89	13	86	86	13	49
**Eye**	129	177	220	57	14	989	43	28	96	43	124	28	67	91	43	0
**Embryonic**	306	701	98	41	0	1304	15	0	67	77	171	5	124	213	5	0
**Abdominal cav.**	**964**	642	98	49	0	2743	0	0	24	24	247	24	98	123	0	0
**Cervix**	537	247	123	103	0	1177	20	0	103	82	123	20	82	103	0	20
**Ovary**	93	409	74	83	0	1581	9	18	46	37	102	0	37	130	55	18
**Uterus**	287	608	721	266	0	2334	125	8	79	41	133	16	95	125	20	8
**Prostate**	153	262	1410	237	6	1653	32	19	44	38	115	32	44	102	32	0
**Testis**	383	252	197	44	67	2222	411	3	140	73	118	**220**	389	130	31	9
**Bladder**	423	195	782	1108	0	1858	0	130	228	97	0	65	195	32	0	0
**Kidney**	349	130	931	214	9	4470	204	9	97	23	69	55	158	274	93	46
**Tongue**	590	132	1388	118	0	1905	162	14	29	103	236	29	280	44	14	0
**Larynx**	132	498	365	66	0	565	0	0	0	66	0	0	66	66	33	0
**Pharynx**	69	139	69	0	0	765	69	0	0	0	0	139	0	69	0	0
**Salivary gl.**	96	0	0	0	0	387	145	0	48	96	145	48	0	48	0	0
**Heart**	246	190	1692	347	11	2129	67	44	33	22	33	11	56	44	33	67
**Lymph node**	165	176	10	10	0	590	0	0	82	217	103	0	176	10	41	0
**Lymph**	334	44	0	0	0	1381	0	0	0	0	89	0	111	111	0	0
**Tonsil**	0	0	115	0	0	463	57	0	0	231	115	0	57	57	0	0
**Spleen**	302	113	**7292**	1416	0	5516	94	75	18	18	18	0	37	56	18	**94**
**Thymus**	279	39	1982	399	0	4444	13	172	53	53	39	13	172	0	0	13
**Mammary gl.**	524	647	647	104	5	2145	17	17	64	52	169	11	81	**1119**	46	0
**Muscle**	393	113	262	52	0	2963	26	8	26	34	87	52	139	157	43	0
**Lung**	247	213	1467	153	8	1166	17	76	48	25	102	22	125	201	14	17
**Trachea**	224	40	2595	797	0	3310	61	**265**	**265**	40	20	163	408	143	0	20
**Skin**	432	173	358	42	0	2861	179	26	21	47	226	0	131	158	21	5
**Umbilical cord**	148	148	0	0	0	1782	**816**	0	0	0	0	0	0	297	0	74
**Vascular**	411	176	1038	58	0	**8247**	19	0	117	0	0	19	176	842	39	0
**Small intestine**	357	447	3914	380	22	4853	357	0	67	22	134	0	134	201	0	0
**Colon**	251	374	320	103	0	2986	9	29	24	39	123	9	118	226	4	4
**Stomach**	126	466	641	145	0	1856	38	29	0	19	**456**	9	204	58	0	0
**Esophagus**	578	1261	2732	**1839**	0	1839	262	157	52	105	105	52	262	683	0	0
**Nerve tissue**	156	509	3725	470	0	1215	39	39	0	39	39	0	196	196	**235**	39

^a^Each number represents the number of Gene ESTs times 10^6 ^over total number of Tissue ESTs, as reported in the NCBI UniGene database (2/2007). Boldface highlights the maximum for each Gene (two very close maxima are highlighted for HSPA12A). Abbreviations: cav., cavity; gl., gland.

We also obtained data on expression of *hsp70 *genes at successive developmental stages (embryo, and less than 4 weeks, 4 weeks to 17 years, and over 17 years of age) (Table 7). The results indicated that most *hsp70 *paralogs are preferentially expressed at specific developmental stages. Seven genes (HSPA8, HSPA1A, HSPA9B, HSPA1B, HSPH1, HYOU1, HSPA12A) were expressed at their highest levels in juvenile tissues (4 weeks to 17 years), three genes (HSPA1L, STCH, HSPA14) were expressed at their highest levels in the embryo, three genes (HSPA2, HSPA6, HSPA12B) were most expressed in the neonate (less than 4 weeks old), only two genes (HSPA5, HSPA4) were most expressed in adult tissue (17 years old and older), and one gene (HSPA4L) was equally expressed, at very low levels, in the embryo and in the adult and was undetectable in the neonate and juvenile tissues.

**Table 7:** Expression levels of human *hsp70 *genes at various stages of development^a^

Gene	Developmental stage			
	
	Embryo	Neonate (<4 weeks)	Juvenile (<17 years)	Adult (>17 years)
HSPA9B	258	597	**798**	276
HSPA5	289	74	272	**418**
HSPA1A	562	523	**1124**	337
HSPA1B	122	149	**235**	106
HSPA1L	**9**	0	0	2
HASPA8	1944	7023	**7384**	2006
HSPA2	23	**410**	36	69
HSPA6	6	**74**	0	30
STCH	**72**	0	0	55
HSPA14	**61**	0	36	47
HSPA4	104	0	72	**144**
HSPA4L	17	0	0	18
HSPH1	163	186	**362**	133
HYOU1	167	0	**526**	142
HSPA12A	26	37	**108**	41
HSPA12B	6	**37**	0	13

^a^Each number represents the number of Gene ESTs times 10^6 ^over total number of Tissue ESTs as reported in the NCBI UniGene database (2/2007). Boldface highlights the maximum for each Gene.

We obtained data on the mode of expression of *hsp70 *genes and the locale of residence of their proteins as shown in Table 8. Ten of the 14 genes studied were expressed constitutively, and out of these 10 genes five were expressed only constitutively (induction by stress was either untested or when tested the results indicated that the gene was not stress inducible). Only one gene was not constitutively expressed, and nine genes were stress inducible.

**Table 8:** Human *hsp70 *genes: Expression modality and location of their proteins

Gene	Cell locale	Tissue	Modality of expression
HSPA8	Cytosol. Translocate to nucleus and nucleoli upon heat shock. Surface of embryonic stem cells	Highly expressed in all tisuses	Constitutive. Moderately induced by heat shock
HSPA2	Nucleus	Mostly in testis, skeletal and heart muscles, esophagous, brain	Constitutive
HSPA1A and HSPA1B	Cytosol. Nucleus and nucleoli upon heat shock	All tissues	Strongly induced by heat shock
HSPA1L	Mostly cytosol under basal conditions. Nucleus but not nucleoli upon heat shock	Spermatides	Constitutive. Not induced by heat shock
HSPA6	Cytosol. Nucleus	Most tissues	Induced by heat shock. No basal expression
HSPA5	Endoplasmic reticulum (ER)	All tissue	Induced by ER stressors
HSPA9B	Mitochondria. ER. Cytosol. Cytosolic vesicles. Membrane surface	Many tissues	Constitutive
STCH	Microsomas	All tissues	Constitutive
HSPA14	Cytosol. Associated to ribosomes	Many tissues (low expression)	Undetermined
HSPA4	Cytosolic (?)	Most tissues	Constitutive. Not induced by heat shock
HSPA4L	Cytosol. Nucleus upon heat shock	Mostly testis	Constitutive. Induced by heat shock in somatic cells but not in germ cells
HSPH1	Cytosol. Nucleus upon heat shock, possibly nucleoli	Most tissues	Constitutive. Induced by heat shock
HYOU1	ER. Cytosol (isoform 3)	Liver, pancreas. Highly expressed in cells with well developed ER	Induced by ER stressors
HSPA12A	Undetermined yet	Brain. Kidney. Muscle	Constitutive. Induced by stress
HSPA12B	Undetermined yet	Skeletal and heart muscles	Constitutive. Induced by stress

## Discussion

### Multiple genes and mRNA variants: Hsp70 diversity

We identified in the human genome 17 genes (Table 1) encoding proteins of the extended Hsp70 family. This count includes one putative gene, HSPA7, transcribed after a 45°C heat shock in fibroblasts [26] but also proposed to be a pseudogene transcribed only under particular conditions [27]. This list considerably expands the count of 11 genes resulting from a "pre-genomic" attempt at a comprehensive classification of human *hsp70 *genes [12] and resizes a recent list of 21 putative genes [11] collected from public databases, which included four pseudogene sequences. At least five genes are characterized by multiple mRNA variants and protein isoforms. We also identified in the human genome 30 *hsp70 *pseudogenes (seven for the first time), most of which (27 pseudogenes) we determined originated from the gene HSPA8.

Our sequence and domain comparisons show that the Hsp70 family is defined by its unique ATP-binding domain (ABD), conserved in all of its members with the exception of the two HSPA12 genes, where the ABD is more divergent but still recognizable. Within the ABD we have identified sequence insertions, most of which uniquely characterize sequences from Group III (HSPA4, HSPH and HYOU1, see Figure 6). These regions are interesting targets for future functional characterization studies. In contrast to the substantial conservation of the ABD, the substrate-binding domain (SBD) has greatly diversified among the various Hsp70 Groups, including complete or partial loss respectively in STCH and HSPA14, significant sequence divergence and insertion of novel sequence elements in Group III genes, and complete domain substitution in the Group I sequences (HSPA12A and HSPA12B). The extent of sequence divergence, exemplified by substitution or loss within the SBD, suggests that the Hsp70-like proteins belonging to Groups I, III, IV, and V are likely to have evolved structures and functions in the SBD domain that are not necessarily associated to the classic hydrophobic peptide-recognition mechanism of the typical Hsp70 sequences of Groups II, VI and VII.

We also characterized presence or absence of protein-protein interaction sequence motifs in different family members, indicating which members can be predicted to participate in certain interaction networks. For example, we found that the TPR1 domain-binding motif [23] (shown as TPR in Figure 6), mediating interaction of Hsp70 with Hsp90, was conserved in all the sequences of Group VI and in HSPA4L, HSPA4 and HSPH1, strongly suggesting a functional relation of these proteins through their interaction with Hsp90. In contrast, the motif was absent in HSPA5, HSPA9B, STCH, HSPA14, HYOU1, HSPA12A, or HSPA12B, suggesting in this respect a different role for these proteins in the human interactome. In a similar vein, we identified in most Hsp70 sequences (excepting only HSPA12A and B) a ubiquitin-binding peptide (shown as UBP in Figure 6) previously described only in STCH [24], suggesting that all typical and the majority of the atypical Hsp70 proteins are involved in the ubiquitin system and protein-degradation pathways.

Analyses of EST data suggest that some *hsp70 *genes may express an impressively high number of mRNA variants and protein isoforms (see for example Table 4). To what extent these variants correspond to proteins with distinct functions and/or disparate mechanisms of action, or to transcriptional errors, remains to be established. In any case, the results indicate that many more *hsp70 *gene transcripts and protein variants than previously suspected may be expressed in human cells. This suggests that the use of alternative forms of gene expression, either through different transcription initiation and/or translation initiation sites or alternative splicing, may be a phenomenon significantly more important than previously recognized in generating the many functionalities of the Hsp70 family in the various tissues and cell compartments. The different functionalities that may be generated through these mechanisms are exemplified by the HYOU1 gene. Two transcript variants of this gene are generated from two alternative transcription-initiation sites corresponding to alternative first exons 1A and 1B of different lengths but with the same translation-initiation site. A 21 bp *cis*-acting element overlaps the 5' end of exon 1A and is involved in hypoxic stress-dependent induction of the alternative transcript that begins with exon 1B, resulting in increased accumulation of the protein in the ER [28]. In a third transcription variant, starting within what is exon 2 of the transcripts mentioned above, a different protein isoform is produced with translation-initiation within exon 3, lacking the ER-import signal peptide. This variant is thought to have housekeeping functions in the cytosol [28]. The elucidation of the significance of the known and predicted mRNA variants of *hsp70 *genes would be of great importance for understanding the various functions of Hsp70 proteins in all cellular compartments, tissues, and developmental stages under diverse environmental conditions.

### Evolution, groups, and subgroups of human Hsp70

Their great diversity notwithstanding, human Hsp70s can be sorted into evolutionarily related clusters. Early analyses based on sequences of eight typical Hsp70 proteins from yeast and other typical Hsp70 proteins from plants and animals, including one from humans, showed that typical *hsp70 *genes cluster in four evolutionary groups that are respectively characterized by expression in cytoplasm, ER, mitochondria or plastids [29]. Our phylogenetic analysis revealed that the 17 human Hsp70s characterized in this work belong to seven major Groups (Figure 1), confirmed by other sequence features, such as "signature sequences" (sequence elements present in one Group but not in others) and exon-intron gene structure, and correlated with other data (e.g., molecular mass and locale of residence). The phylogenetic tree of the human Hsp70s of about 70 kDa molecular weight unveiled three major evolutionary events: firstly, two genes with products of molecular weight around 70 kDa converged into a primordial eukaryotic organism, one derived from an Alphaproteobacterium, from which HSPA9B of mitochondrial origin derived, and one, of unrecognized prokaryotic origin, corresponding to the nuclear gene progenitor of HSPA8 and HSPA5; secondly, the nuclear gene most likely first duplicated into two genes, one specialized to the cytosol/nucleus (orthologous to HSPA8), and one specialized to the ER (orthologous to HSPA5); thirdly, the gene specialized to the cytosol/nucleus later multiplied into a multigene family (Group VI) represented in humans by six or seven genes (HSPA8, HSPA2, HSPA1A, HSPA1B, HSPA1L, HSPA6 and possibly HSPA7) and at least 27 pseudogenes (Figures 1 and 2, and Tables 2 and 6).

Within Group VI, only the coding region of HSPA8 was encoded by multiple exons (two isoforms encoded by eight and seven exons, respectively), whereas the coding regions of all other genes are encoded within a single exon (although HSPA2 and HSPA1L each possesses a 5' untranslated exon). Similarly, most pseudogenes associated to HSPA8 did not show signs of exon-intron structures. This suggests that the sequences of group VI and related pseudogenes were all derived from HSPA8 by retrotransposition. The impressive retrotransposition activity (perhaps L1 associated) involving HSPA8 is also consistent with the very high level of expression of this gene in comparison with the other members of the *hsp70 *family. The multi-exon structure of all other genes suggests instead that sets of similar sequences (e.g., the HSPA4 and HSPA12 subgroups) were generated by duplication events.

The evolutionary history of HSPA1A, HSPA1B and HSPA1L has been analyzed in detail in the past [30] and revisited more recently [31]. The three genes are clustered with the same organizational structure in the human and mouse genomes, strongly suggesting that their duplication happened before the separation of the rodent and primate lineages. The phylogenetic trees, however, suggest that HSPA1A and HSPA1B resulted from independent duplications in human and mouse. This apparent contradiction can be interpreted to be the result of gene transformation between HSPA1A and HSPA1B occurring independently in human and mouse.

The human HSPA6 and HSPA7 sequences do not significantly cluster with any other animal protein. This result is consistent with an early separation of HSPA6/7 and the common progenitor of HSPA8, HSPA2 and HSPA1, probably predating the evolution of Vertebrates. The presence of sequences from Fish and Amphibians in the lineage of HSPA8 and the absence of a sequence from Fish in the lineages of HSPA1 and HSPA2 suggests that HSPA1 and HSPA2 originated from HSPA8 early in the evolution of the tetrapod lineage, with the exclusion of Fish, although homologs of these genes might be found in *Danio rerio *among the many *hsp70 *genes of unresolved origin.

Less well defined is the origin of the atypical human Hsp70-like proteins, comprising two lighter sequences (about 60 kDa), HSPA14 and STCH, the first associated to ribosomes and the second expressed in microsomes, and of the heavier 105/110 kDa HSPA4, HSPA4L, HSPH1 proteins and 150 kDa HYOU1 protein (the latter also localized to the ER). However, the greater similarity of these sequences to the typical human Hsp70 product compared to the prokaryotic DnaK proteins suggests that these genes also originated from a common progenitor of the typical nuclear eukaryotic genes from a gene duplication that predated the proliferation of the *hsp70*-typical gene sequences. Also uncertain is the evolutionary position of HSPA12A and HSPA12B, which conserve only sparse similarity to the ATP-binding domain of typical human Hsp70 proteins, and are marginally more similar to prokaryotic DnaK [16]. We found that the evolutionary relations of these multiexon sequences are not clarified by their gene structure. In fact, whereas the positions of the splice sites within each Group were generally conserved, we found very few conserved splice sites when comparing sequences from different Groups.

## Conclusion

The human *hsp70*-gene family is in the main the result of multiple duplications facilitated by frequent retrotransposition events of a single highly expressed gene, HSPA8. Human Hsp70 proteins can be clustered into seven distinct evolutionary Groups, many of which are characterized by highly divergent C-terminal (substrate-binding) domains. Hence, functional specificities of the various Hsp70 proteins are likely to be associated to the functional properties of their C-terminal domains. In addition, other noticeable differences in the nucleotide-binding N-terminal domain of the various Hsp70s may also affect the functional role of these proteins.

Overall, the results revealed an evolutionary diversity at genetic, transcriptional, and post-transcriptional levels that generated a multiplicity of structures, functions, regulation modes, and anatomic locations of Hsp70s, predicting a wide array of roles in cell physiology and pathogenesis.

A thorough classification of all the members of the *hsp70 *gene family encoded in the genome is an essential step towards their unambiguous functional characterization. The identification of the chaperonopathies [5-7,32,33] has made more pressing the need for a better characterization of these genes in order to pinpoint with accuracy the gene-proteins involved, understand pathogenetic mechanisms, and devise diagnostic and therapeutic means. Bioinformatics analyses of evolutionary relations and sequence features of the numerous *hsp70 *paralogs found in the human genome suggest many interesting avenues of experimental studies (e.g., to determine specific functions in specific tissues of any given Hsp70) and provide the basis for selecting the most efficient molecule for use in chaperonotherapy or the most appropriate target (Hsp70 molecule and/or segment therein) for anti-chaperone agents.

## Methods

### Chaperonomics

The strategies and methods described previously for chaperonomics [31] were applied throughout. Chaperonomics encompasses a series of concatenated, complementary methods that are applied in sequence to obtain layer after layer of results that progress toward the building of a detailed picture of genes and their proteins, including structural and evolutionary information at various levels.

### Gene identification

*hsp70*-like DNA sequences were identified as matches to 13 distinct reference-Hsp70 query-sequences (proteins) selected from a much larger pool of sequences available in public databases, mining the NCBI reference human genome sequence Build 36.1 (2006) using the search engines BLAT [34], at the University of California at Santa Cruz (UCSC), and BLAST [35], at NCBI (TBLASTN for Protein to DNA sequence). The DNA strand and chromosomal location were recorded as per the UCSC genomic browser.

### Exon and intron identification

Initially, intron, exons, and alternative spliced isoforms were recorded as reported in the gene description pages from the NCBI "Entrez Gene" annotation and from the UCSC Genome Browser annotation links. We also carried out literature searches to identify some of the variants (e.g., for HYOU1). As a last step, we verified all intron/exon structures by aligning the protein and processed mRNA sequences to the genomic sequence, using the BLAT [34] search engine, identifying introns as genome sequences interspersed between aligned regions (exons).

### Identification and characterization of diverged or degenerate sequences

Diverged or degenerate sequences were identified with the BLAST search engine, enabling us to collect and evaluate sequence regions that displayed similarity to *hsp70 *genes of lower significance. For each BLAST hit (aligned segment), we extracted the flanking genome sequence up to several thousand nucleotides 5' and 3' of the hit. We then translated the sequences encoded in the three frames of the strand where the hit was identified and, subsequently, we aligned the three translations to the query Hsp70 proteins using ITERALIGN [36] and, by visual inspection of the alignments, we identified all conserved Hsp70-like sequence regions linked by short intervening sequences. When regions proximal to the 5' end of the query genes were conserved, the sequence was extended up to the proximal Start codon (ATG) in the approximately expected position, when present; otherwise, the sequence was initiated at the first conserved region. Similarly, the sequence was extended 3' of the last conserved segment up to the fist stop codon, when present, or else terminated at the last conserved segment. The predicted sequence was matched to the human genome using BLAT. Long genome sequences interspersed between the aligned portions (predicted as exons) of the query sequence were considered potential introns.

The sequence assembly process described above enabled us to obtain a complete characterization of the patterns of conservation for these sequences, and to identify indicators of degeneracy in the coding region, such as frame-shifts (locations where the alignment in one frame of translation switched to an alignment in a different frame of translation) and in-frame stop codons (i.e., stop codons within a conserved *hsp70*-like sequences).

### Identification of other eukaryotic, nonhuman *hsp70 *genes

We collected *hsp70 *genes and Hsp70 proteins from nonhuman eukaryotic organisms for which complete genome sequences are available, by querying the respective genome sequences with the protein similarity search program SSPA [36].

### Protein similarity

All similarities reported are SSPA (Significant Segment Pair Alignment) similarities [36] determined by using the Blosum62 [37] amino acid similarity matrix.

### Protein isoform identification

Many protein/mRNA isoforms were recorded as listed in the annotations from the UCSC BLAT server Browser pages and in the NCBI Entrez Gene pages. In addition, we identified other variants by comparing sequences and through literature searches.

### Phylogenetic analysis

Evolutionary analyses were performed using protein sequences from the *hsp70 *genes collected from the human genome and other eukaryotic genomes and pseudo-translations of human *hsp70 *pseudogenes. We excluded all sequences of length less than 300 aa (labeled as "fragment") or too diverged to produce reliable alignments. We performed the alignments with Clustal W and constructed evolutionary trees using the neighbor-joining algorithm [38] with the Kimura transformation for protein sequences [39], and with bootstrap analyses based on 1000 samples. For evolutionary trees of human sequences we repeated the analyses using the maximum-likelihood method implemented in PHYML [40].

### Sources of gene structure and expression data based on EST and SAGE analyses

Predictions of gene structure variants were collected and organized from the ECgene web-site [43] and database [14,15] and from the NCBI AceView database [41], based on EST and SAGE expression data. Expression data based on the number of tissue-specific and developmental-stage-specific ESTs were collected from the NCBI UniGene database [25].

## Abbreviations

ABD, ATP-binding domain; SBD, substrate-binding domain; UBP, ubiquitin-binding peptide; TPR1, tetratrico peptide repeat 1; ER, endoplasmic reticulum

## Authors' contributions

All authors contributed equally to this work in discussing research strategy and development, in data collection and classification, and in writing the manuscript. LB performed the bioinformatics analyses. All authors read and approved the final manuscript.

## Supplementary Material

Additional file 1 Figures S1–S4 and Table S1. Alignments of Hsp70 sequences with splice-site positions (Figures S1–S3), evolutionary tree of eukaryotic typical *hsp70 *genes (Figure S4), and genome position and length of each exon of *hsp70*-like genes (Table S1). Click here for file
