# Supplementary material for: hsp70 genes in the human genome: Conservation and differentiation patterns predict a wide array of overlapping and specialized functions
Source: BMC Evol Biol. 2008 Jan 23;8:19. doi: 10.1186/1471-2148-8-19 (PMC2266713; doi:10.1186/1471-2148-8-19)
Supplement: Additional file 1 — Figures S1–S4 and Table S1. Alignments of Hsp70 sequences with splice-site positions (Figures S1–S3), evolutionary tree of eukaryotic typical hsp70 genes (Figure S4), and genome position and length of each exon of hsp70-like genes (Table S1). [file 1471-2148-8-19-S1.pdf]

Luciano Brocchieri, Everly Conway de Macario, and Alberto J. L. Macario

hsp70 genes in the human genome: conservation and differentiation patterns predict a wide array of overlapping and specialized functions

Additional file 1

File format: PDF

Title: Figures S1-S4 and Table S1

Description: Alignments of Hsp70 sequences with splice-site positions (Figures S1-S3), evolutionary tree of eukaryotic typical *hsp70* genes (Figure S4), and genome position and length of each exon of *hsp70*-like genes (Table S1).

Additional file 1 figure legends

Figure S1. Alignment of human Hsp70 sequences belonging to Groups II, VI, and VII with the position of splice site indicated by red numbers. The number indicates the base (1, 2, or 3) of the codon corresponding to the amino acid residue preceding the number in the alignment. The alignment was obtained using the multiple alignment program ITERALIGN [36], with aligned residues shown in upper case and residues not aligned by the program shown in lower case. For each line, the sequence positions of the first and last residues are indicated.

Figure S2. Alignment of Hsp70 sequences belonging to Groups III with the position of splice site indicated by red numbers. See legend for ***Figure S1, Additional File 1*** for explanations.

Figure S3. Alignment of Hsp70 sequences belonging to all seven evolutionary Groups shown in *Figure 1*. See legend for ***Figure S1, Additional File 1*** for explanations.

Figure S4. Evolutionary tree of eukaryotic typical *hsp70* genes

Included are the proteins encoded in the typical human *hsp70* genes (Groups II, VI, and VII in ***Figure 1***) and in the *hsp70* genes of eighteen other completely sequenced eukaryotic genomes. Sequences from the latter nonhuman eukaryotic genomes were identified using as queries human proteins from Groups II, VI, and VII. See legends to ***Figures 1*** and ***3*** and text for details on methods and calculation of bootstrap values. Branches supported by bootstrap values of less than 40% were collapsed in the tree representation. Human proteins are in red. DnaK (*E. coli*) is the Hsp70 sequence AAC73125.1 from *E. coli*. Acronyms in black indicate the eukaryotic genomes in which other *hsp70* genes were found, as follows: ANOGA: *Anopheles gambiae* (Insects); ARATH: *Arabidopsis thaliana* (Plants); CANGL: *Candida glabrata* (Fungi); CAEEL: *Caenorhabditis elegans* (Nematoda); CHICK: *Gallus gallus* (Birds); CYAME: *Cyanidioschyzon merolae* (Red alga); DANRE: *Danio rerio* (Fish); DROME: *Drosophila melanogaster* (Insects); ENCCU: *Encephalitozoon cuniculi* (Fungi, Microsporidia); ENTHI: *Entamoeba histolytica* (Protists); GIALA: *Giardia lamblia* (Protists); LEIMA: *Leishmania major* (Protists); MOUSE: *Mus musculus* (Mammals); NEUCR: *Neurospora crassa* (Fungi); PLAFA: *Plasmodium falciparum* (Protists); SCHPO: *Schizosaccharomyces pombe* (Fungi); TRYCR: *Trypanosoma cruzi* (Protists); XENTR: *Xenopus tropicalis* (Amphibians); YEAST: *Saccharomyces caerevisiae* (Fungi).

Figure S1

```
HSPA9B           1 misasraaaarlvgaaasrgptaarhq 3 dswnglsheafrlvsrrdya 2 s  48
HSPA8-1          1 MSK                                                     3
HSPA8-2          1 MSK                                                     3
HSPA5            1 mklslvaamllllsaaraeeedkkedv                            27


HSPA9B          49 eaik GAVVGIDLGTTNSC   VAVME GKrAK 3 VLENAEGARTTPSVVA  92
HSPA8-1          4      GPAVGIDLGTTYSC   VGVFQHGK VE   IIANDQGNRTTPSYVA  43
HSPA8-2          4      GPAVGIDLGTTYSC   VGVFQHGK VE   IIANDQGNRTTPSYVA  43
HSPA5           28      GTVVGIDLGTTYSC 2 VGVFKNGR VE   IIANDQGNRITPSYVA  67


HSPA9B          93 FTADGERLVGMPAKRQ AVTNPNNTFYA   TKRLIGRRYDDPEVQKDI 2   137
HSPA8-1         44 FTDT ERLIGDAAKNQvAM NPTNTVFD 1 AKRLIGRRFDDAVVQSDM     87
HSPA8-2         44 FTDT ERLIGDAAKNQvAM NPTNTVFD 1 AKRLIGRRFDDAVVQSDM     87
HSPA5           68 FTPEGERLIGDAAKNQ LTSNPENTVFD   AKRLIGRTWNDPSVQQDI     112


HSPA9B         138 KNVPFK   IV r  ASNGDAWVE ahg      KLYSPSQIGAFVLMKMKE  176
HSPA8-1         88 KHWPFM   VV ND AGRPKVQVE YK  GET  KSFYPEEVSSMVLTKMKE  129
HSPA8-2         88 KHWPFM   VV ND AGRPKVQVE YK  GET  KSFYPEEVSSMVLTKMKE  129
HSPA5          113 KFLPFK 3 VV ek KTKPYIQVD igg GQT  KTFAPEEISAMVLTKMKE  155


HSPA9B         177 TAE 1 NYLGR    TTAKNAVITVPAYFNDSQRQ 3 ATKDAGQISGLNVLRV  219
HSPA8-1        130 IAE   AYLGK 3  TTVTNAVVTVPAYFNDSQRQ   ATKDAGTIAGLNVLRI  172
HSPA8-2        130 IAE   AYLGK 3  TTVTNAVVTVPAYFNDSQRQ   ATKDAGTIAGLNVLRI  172
HSPA5          156 TAE   AYLGKk 3   VTHAVVTVPAYFNDAQRQ   ATKDAGTIAGLNVMRI  198


HSPA9B         220 INEP   TAAALAYGLDKS   e   DKv 2 IAVYDLGGGTFDISILEIQKG  260
HSPA8-1        173 INEP   TAAAIAYGLDKK 3 VGA ER   NVLIFDLGGGTFDVSILTIEDG  215
HSPA8-2        173 INEP   TAAAIAYGLDKK 3 VGA ER   NVLIFDLGGGTFDVSILTIEDG  215
HSPA5          199 INEP 2 TAAAIAYGLDKR   eg  EK   NILVFDLGGGTFDVSLLTIDNG  240


HSPA9B         261 VFEVKSTNGDTFLGGEDFDQALLRHIVKEFKRE 3 TGVDLTKDNMALQR  307
HSPA8-1        216 IFEVKSTAGDTHLGGEDFDNRMVNHFIAEFKRK   HKKDISENKRAVRR  262
HSPA8-2        216 IFEVKSTAGDTHLGGEDFDNRMVNHFIAEFKRK   HKKDISENKRAVRR  262
HSPA5          241 VFEVVATNGDTHLGGEDFDQRVMEHFIKLYKKK   TGKDVRKDNRAVQK  287


HSPA9B         308 VREAAEKAKCELSSSVQ                    3 tdinlpyltmds  336
HSPA8-1        263 LRTACERAKRTLSSSTQ  ASIEIDSLYEGIDFYTS                 296
HSPA8-2        263 LRTACERAKRTLSSSTQ  ASIEIDSLYEGIDFYTS                 296
HSPA5          288 LRREVEKAKRALSSQHQ  ARIEIESFYEGEDFSET                 321


HSPA9B         337 sgpkhlnmk LTRAQFEGIVT   DLIRRTIAPCQKAMQDAEVSKSDIGE  382
HSPA8-1        297           ITRARFEELNA   DLFRGTLDPVEKALRDAKLDKSQIHD  333
HSPA8-2        297           ITRARFEELNA   DLFRGTLDPVEKALRDAKLDKSQIHD  333
HSPA5          322           LTRAKFEELNM 3 DLFRSTMKPVQKVLEDSDLKKSDIDE  358
```

Figure S1 (continued 1)

```
HSPA9B          383 VILVGGMTRMPK 3 VQQTVQDLF GRAPSKAVNPDEAVAIGAA   IQGGV  427
HSPA8-1         334 IVLVGGSTRIPK   IQKLLQDFFNGKELNKSINPDEAVAYGAA 1 VQAAI  378
HSPA8-2         334 IVLVGGSTRIPK   IQKLLQDFFNGKELNKSINPDEAVAYGAA 1 VQAAI  378
HSPA5           359 IVLVGGSTRIPK   IQQLVKEFFNGKEPSRGINPDEAVAYGAA   VQAGV  404


HSPA9B          428 LAGD vt       DVLLLDVT   PLSLGIETLGGVFTKLINRNTTIPTKK  468
HSPA8-1         380 LSGD KSENVQ   DLLLLDVT   PLSLGIETAGGVMTVLIKRNTTIPTKQ  424
HSPA8-2         380 LSGD KSENVQ   DLLLLDVT   PLSLGIETAGGVMTVLIKRNTTIPTKQ  424
HSPA5           405 LSGD qdtg   1 DLVLLDVC   PLTLGIETVGGVMTKLIPRNTVVPTKK  447


HSPA9B          469 SQ 3 VFSTAADGQTQVEIKV   CQG   EREMAGDNKLLGQFTL 3 i         506
HSPA8-1         424 TQ   TFTTYSDNQPGVLIQV 3 YEG   ERAMTKDNNLLGKFEL   t         462
HSPA8-2         424 TQ   TFTTYSDNQPGVLIQV 3 YEG   ERAMTKDNNLLGKFEL   tg 1 mpg  466
HSPA5           448 SQ   IFSTASDNQPTVTIKV   YEG 1 ERPLTKDNHLLGTFDL   t         485


HSPA9B          507  GIP  PAPRGVPQIEVTFDIDANGIVHVSAKDKGTGREQQI1VIQSSG    551
HSPA8-1         463  GIP  PAPRGVPQIEVTFDIDANGILNVSAVDKSTGKENKITITNDKG 1  508
HSPA8-2         467  GMP                                                469
HSPA5           486  GIP  PAPRGVPQIEVTFEIDVNGILRVTAEDKGTGNKNKITITNDQN    531


HSPA9B          552   GLSKDDIENMVKNAEKYAEEDRRKK 3 ERV EAVNMAEGIIH dtetkm  596
HSPA8-1         509   RLSKEDIERMVQEAEKYKAEDEKQR   DKVSSKNSLESYAFN mkatve  554
HSPA8-2         470 g                                             gfpggg  476
HSPA5           532   RLTPEEIERMVNDAEKFAEEDKKLK   ERIDTRNELESYAYS lknqig  577


HSPA9B          597   EEFKDQLPADE 3 CNKLKEEISKMRELLARKD   SE tg ENIRQAASSLQ  641
HSPA8-1         555   DEKLQGKINDE  DKQKILDKCNEIINWLDKNQ 3 TA ek EEFEHQQKELE  600
HSPA8-2         477 a                                       pp               479
HSPA5           578 d KEKLGGKLSSE  DKETMEKAVEEKIEWLESHQ   DA di EDFKAKKKELE  624


HSPA9B          642 QA sl KLFEMAYKK 3 maseregsgssgtgeqkedqkeekq          679
HSPA8-1         601 KV cn PIITKLYQS   aggmpggmpggfpgggappsggassgptieevd  646
HSPA8-2         480    sg             gassgptieevd                       493
HSPA5           625 EI vq PIISKLYGS   agppptgeedtaekdel                  654
```

Figure S2

```
HSPA4L           1                                         MSVVGIDLG flncy   14
HYOU1-1          1 madkvrrq   rprrrvcwalvavlladllalsd 1 t LAVMSVDLG sesmk   46
HSPA14-a         1                                         MAAIGVHLG ctsac   14
STCH             1 maremtil 1 gsavltlllagylaqqylplpt       PKVIGIDLG ttycs   44

HSPA4L          15 IAVAR    SG    G IETIANE   ysdrc  TP 2 ACISL g SRTRA   I G na  51
HYOU1-1         47 VAIVK    PG v  P MEIVLNK 2 esrrk  TP   VIVTL   KENER   FFG ds  84
HSPA14-a        15 VAVYK 3 DG ra G    VVAND   agdrv  TP   AVVAY   SENEE 3 IVG l   50
STCH            44 VGVFF    PG t  GkVKVIP D   enghis IP   SMVSF   TDNDV   YVG ye  83

HSPA4L          52  AKSQ 3 i      VTNVRNTI hgf KKLHGRS   FDDPIVQ teri RLP y  89
HYOU1-1         85  AASM 3 a      IKNPKATL ryf QHLLGKQ   ADNPHVA lyqa RFP e  122
HSPA14-a        51  AAKQ   sr     IRNISNTV mkv KQILGRS 2 SSDPQAQ kyia     e  86
STCH            84         svela DSNPQNTI cda KRFIGKI   FTAEELE aeig RYP f  121

HSPA4L          90 e   lqk   mpngsag VK 3 VRY LE   EE rp FA IEQVtG  MLLAKLKE   TSE   130
HYOU1-1        123 h   elt   fdpqrqt VH   FQI SS 2  Q lq F SPEEVlG  MVLNYSRS   LAE   162
HSPA14-a        87 s   kcl 3 vieknqk LR   YEIdTG   EE tk FVNPEDV A RLIFSKMKE 1 TAH   129
STCH           122 k 3 vl    nkngm   VE   FSV TS   NE ti TVSPEYV GsRLLL KLKE   MAE   160

HSPA4L         131 nalk   k PVADCVIS 3 IPSF FTDAERRSVM   AAAQVA   GLNCLRLMNE  173
HYOU1-1        163 dfae 1 q PIKDAVIT   VPVF FNQAERRAVL   QAARMA   GLKVLQLIND  205
HSPA14-a       130 svlg   s DANDVVIT   VP FdFGEKQKNALG 2 EAARAA   GFNVLRLIHE  172
STCH           161 aylg   m PVANAVIS   VPAE FDLKQRNSTI   EAANLA 1 GLKILRVINE  203

HSPA4L         174 TTAV 1 ALAYGI  YKQ DL ppldekpr    NVVFIDMG HSAYQVLVCAF  215
HYOU1-1        206 NTAT   ALSYGV  FRRkDI nttaq     3 NIMFYDMGsGSTV CTIVTY  245
HSPA14-a       173 PSAA   LLAYGI         gqdsptgks 2 NILVFKLG GTSLSLSVMEV  210
STCH           204 PTAA   AMAYGL  HKA DV f           HVLVIDLG GGTLDVSLLNK  238

HSPA4L         216 NKGKLK 3 vlat   tf               DPYLGG rnfd      EALVDYFC  245
HYOU1-1        246 Q   MVK  tkea   gmqpqlqirgvg 2 f DRTLGG lemelrlr ERLAGLFN  288
HSPA14-a       211 NSGIYR   vlst   nt               DDNIGG ahft      ETLAQYLA  240
STCH           239 QGGMFL   tram 1 sg               NNKLGG qdfn      QRLLQYLY  268

HSPA4L         246 DE fkt   kyki NVKENSRALLRLYQECEKLK kl MSANAS dlpl   niec  290
HYOU1-1        289 EQ rkg   qrak DVRENPRAMAKLLREANRLK tv LSANAD hmaq 3 ieg   332
HSPA14-a       241 SE fqr 2 sfkh DVRGNARAMMKLTNSAEVAK hs LSTLGS ancf   pds   284
STCH           269 KQ iyq   tygf                        vp        srke   eihr  287

HSPA4L         291  FMNDLD                                                 296
HYOU1-1        333  LMDDVD                                                 338
HSPA14-a       285  LYEGQD                                                 290
STCH           288         lrqavemvklnltlhqsaqlsvlltveeqdrkephssdtelp      329
```

Figure S2 (continued 1)

```
HSPA4L         297                                  VSSKMNR 2 aq FEQLCA  311
HYOU1-1        339                                  FKAKVTR   ve FEELCA  353
HSPA14-a       291                                  FDCNVSR 2 ar FELLCS  305
STCH           330 kdklssaddhrvnsgfgrglsdkksgesqvl FETEISR   kl FDTLNE  375


HSPA4L         312 SLLARVEP PLKAVMEQ an 1 lqr   EDISS   IEIVGGATRIPAVKEQITKF  357
HYOU1-1        354 DLFERV PgPVQQALQS ae   msl 3 DEIEQ   VILVGGATRVPRVQEVLLKA  399
HSPA14-a       306 PLFNKCLE AIRGLLDQ ng   fta   DDINK 3 VVLCGGSSRIPKLQQLIKDL  351
STCH           376 DLFQKILV PIQQVLKE gh   lek   TEIDE   VVLVGGSTRIPRIRQVIQEF  421


HSPA4L         358 F lk     DISTTLNADEAVARGCALQ 3 cai  LSPAFKVREF s ITDLV  398
HYOU1-1        400 V gk 2 e ELGKNINADEAAAMGAVYQ   aaa  LSKAFKVKPF v VRDAV  441
HSPA14-a       352 F pav    ELLNSIPPDEVIPIGAAIE   agil             i        379
STCH           422 F gk     DPNTSVDPDLAVVTGVAIQ   agid             g        448


HSPA4L         399  p YSI     tlrwktsfedgsg 2 ecevfcknh   papfskvitfhkkepfelea   444
HYOU1-1        442  v YPI l 3 veftreveeep     gihslkhnk   rvlfsrmgpypqrkvitfnry  487
HSPA14-a       380  g         kenllvedslmie   csardilvk 3 gvdesgasrftvlfpsgtplp  423
STCH           449  g         swplqvsaleipn   khlqktnfn                          471


HSPA4L         445 fytnlhevpypdarig 1 sftiqn   vfpqsdgdsskvkvk   vrvnihgifsva   493
HYOU1-1        488 shdfnfhinygdlgfl   gpedlr 2 vfgsqnlttvklkgv   gdsfkkypdyes   536
HSPA14-a       424 arrqhtlqapgsissv   clelye   sdgknsakeetkfaq 3 vvlqdldkkengl  473
STCH


HSPA4L         494 sasviekqnle   gdhsdapme   tetsfknenkdnm 3 dkmqvdq   eeghqkcha  542
HYOU1-1        537 kgikahfnlde   sgvlsldr 3 vesvfetlvedsae   eestltk 1 lgntissl   584
HSPA14-a       474 rdilavltmkr 2 dgslhvtct   dqetgkceaisie   ias                  509
STCH


HSPA4L         543 ehtpeeeidhtgaktk 3 sav   sdkqdrlnqtlkkgk                  576
HYOU1-1        585 fgggttpdakengtdt   vq 3 eeeespaegskdepgeqvelkeeaeapvedg  633
HSPA14-a
STCH


HSPA4L         577
HYOU1-1        634 sqppppepkgdatpegekatekengdkseaq 3 kpsekaeagpegvapape  682
HSPA14-a
STCH


HSPA4L         577               VKSIDLPI qsslcrq LGQD llnsyiene 3 gk M  607
HYOU1-1        683 gekkqkparkrrm VEEIGVEL vvldlpd LPED klaqsvqk 2 lqd L  726
HSPA14-a
STCH
```

Figure S2 (*continued 2*)

```
HSPA4L        608 IMQD kl EKERNDAKNAVEEYVYDFR   D rlgtvyekf ITPE 3 dls   649
HYOU1-1       727 TLRD le KQEREKAANSLEAFIFETQ 3 D klyqpeyqe VSTE   eqre  769
HSPA14-a
STCH

HSPA4L        650 KLS avledten WLYEDG edqpkqvy  VDK lqelk 3 kyg  QPI qmk  691
HYOU1-1       770 EIS gklsaast WLEDEG vgattv 3 mLKE klael   rklc QGL ffr  811
HSPA14-a
STCH

HSPA4L        692 ym EHEE r PKALN dlgkkiqlvmkviea  yrnk 3 derydhldptemek  736
HYOU1-1       812 ve ERKK w PERLS aldnllnhssmflk 2 garl   ipemdqiftevemtt 856
HSPA14-a
STCH

HSPA4L        737  VEKCISDAM   SW lnskmnaqnkls L TQD pvvk VSE ivaksk 3 el  778
HYOU1-1       857  LEKVINETW 3 AW knatlaeqak   LpATE kpvl LSK dieakm   mal 898
HSPA14-a
STCH

HSPA4L        779 dnfcnp IIYKPK PKAEvPEDKPK ansehng PMDGQSGTE tksdst  823
HYOU1-1       899 drevqy LLNKAKfTKPR PRPKDK ngtraep PLNASASDQ gekvip  943
HSPA14-a
STCH

HSPA4L        824 kds   sqhtkssgemevd                                     839
HYOU1-1       944 pag 1 qtedaepisepekvetg 1 sepgdteplelggpgae 1 peqkeqstgq  990
HSPA14-a
STCH

HSPA4L
HYOU1-1       991 krplkndel  999
HSPA14-a
STCH
```

Figure S3

```
HSPA9B           1  misasraaaa           rlvg   aaasrgptaarhq 3 dswn     gl 33
HSPA8-1          1  mskgp                                                   5
HSPA5            1  mklslv       AAMLLLL saar   aeeedkkedvg                 28
HSPA4L           1  m                                                       1
HYOU1-1          1  madkvrrqrp           rrrv   cwalvavlladll   ALSD 1 T l  33
HSPA14-a         1  m                                                       1
STCH             1  maremtilg  1 SAVLTLL lagy   laqqylplptp                 31
HSPA12A          1  madkeaggsd           gpre 1 taptsaysspar    SLGD   T gi 33

HSPA9B          34 sheafrlvsrrdya 2 seaikg       AVVGIDLGTT  YSC   VAVME    G k  73
HSPA8-1          6                               A VGIDLGTT  YSC   VGVFQ   HG    24
HSPA5           29                               TVVGIDLGTT  YSC 2 VGVFK   NG    48
HSPA4L           2                               SVVGIDLGFL  NCY    IAVAR  SG    21
HYOU1-1         34                               AVMSVDLGSE  SMK   VAIVK   PG v  54
HSPA14-a         2                               AAIGVHLGCT  SAC   VAVYK 3 DG r  22
STCH            32                               KVIGIDLGTT  YCS   VGVFF   PG t  52
HSPA12A         34 tplspshiv 3 nd   tdsnvseqqsfl VVVAVDFGTT                      s  67

HSPA9B          74          RAK 3 VLENA   EGARTTP   SVVAFTaDGER   LVGMPAKRQ    108
HSPA8-1         25          KVE   IIAND   QGNRTTP   SYVAFT DTER   LIGDAAKNQ    58
HSPA5           49          RVE   IIAND   QGNRITP   SYVAFTpEGER   LIGDAAKNQ    83
HSPA4L          22          GIE   TIANE   YSDRCTP 2 ACISLG SRTR   AIGNAAKSQ 3  55
HYOU1-1         55          PME   IVLNK 2 ESRRKTP   VIVTLK ENER   FFGDSAASM 3  88
HSPA14-a        23 ag             VVAND   AGDRVTP   AVVAYS ENEE 3 IVGLAAKQS    55
STCH            53 g        KVK   VIPDE   NGHISIP   SMVSFT DNDV   YVGYESVEL    87
HSPA12A         68 sgyaysft K E                                                77

HSPA9B         109    AVTNPNNTFYA   TKRLIGRR   YDDPEVQKDI 2 KNVPFK   ivrasn   gdaw   153
HSPA8-1         59 v  AM NPTNTVFD 1 AKRLIGRR   FDDAVVQSDM   KHWPF    mvvnda   grpkv  103
HSPA5           84    LTSNPENTVFD   AKRLIGRT   WNDPSVQQDI   KFLPFK 3 vvekkt   kpy    127
HSPA4L          56    IVTNVRNTIHG   FKKLHGRS   FDDPIVQTER   IRLPY    elqkmp   ngsag  100
HYOU1-1         89    AIKNPKATLRY   FQHLLGKQ   ADNPHVALYQ   ARFPE    heltfd   pqrqt  133
HSPA14-a        56    RIRNISNTVMK   VKQILGRS 2 SSDPQAQKYI   AESKCl 3 viekn    gklry  100
STCH            88    ADSNPQNTICD   AKRFIGKI   FTAEELEAEI   GRYPFK 3 vlnkng   mve    131
HSPA12A         78 pe                                                cihvmr 2 rweg   89

HSPA9B         154 ve   ahg         KLYSPSQIGAFVLMKMKE   TAE 1 NYLGR   t        AKNA   189
HSPA8-1        104 qv   eykget      KSFYPEEVSSMVLTKMKE   IAE   AYLGK 3 t        VTNA   142
HSPA5          128 iq   vdiggg  qt  KTFAPEEISAMVLTKMKE   TAE   AYLGK   k  3     VTHA   168
HSPA4L         101 vk 3 vryle   ee  RPFAIEQVTGMLLAKLKE   TSE   NALKK   p        VADC   140
HYOU1-1        134 vh   fqiss 2 q   LQFSPEEVLGMVLNYSRS   LAE   DFA E 1 qp       IKDA   172
HSPA14-a       101 ei   dtgee   t   KFVNPEDVARLIFSKMKE 1 TAH   SVLGS   d        ANDV   139
STCH           132 fs   vtsne   t   ITVSPEYVGSRLLLKLKE   MAE   AYLGM   p        VANA   170
HSPA12A         90 gd   pgvsn   qkt                                    pttil          104
```

Figure S3 (*continued 1*)

```
HSPA9B         190
HSPA8-1        143
HSPA5          169
HSPA4L         141
HYOU1-1        173
HSPA14-a       140
STCH           171
HSPA12A        105 ltperkfhsfgyaardfyhdldpneakqw   lylekfkmklhttg 3 dltmdt     153


HSPA9B         190                                                         VI  191
HSPA8-1        143                                                         VV  144
HSPA5          169                                                         VV  170
HSPA4L         141                                                         VI  142
HYOU1-1        173                                                         VI  174
HSPA14-a       140                                                         VI  141
STCH           171                                                         VI  172
HSPA12A        154 dltaangkkvkaleifayalqyfkeqalk 3 elsdqagsefensd   vrw    VI  201


HSPA9B         192 T   VPAYFNDSQRQ 3 ATK   DAG Q   ISG   LNVLRVINEP   TAA   ALA  229
HSPA8-1        145 T   VPAYFNDSQRQ   ATK   DAG T   IAG   LNVLRIINEP   TAA   AIA  182
HSPA5          171 T   VPAYFNDAQRQ   ATK   DAG T   IAG   LNVMRIINEP 2 TAA   AIA  208
HSPA4L         143 S 3 IPSFFTDAERR   SVM   AAA Q   VAG   LNCLRLMNET   TAV 1 ALA  180
HYOU1-1        175 T   VPVFFNQAERR   AVL   QAA R   MAG   LKVLQLINDN   TAT   ALS  212
HSPA14-a       142 T   VPFDFGEKQKN   ALG 2 EAA R   AAG   FNVLRLIHEP   SAA   LLA  179
STCH           173 S   VPAEFDLKQRN   STI   EAA N   LAG 1 LKILRVINEP   TAA   AMA  210
HSPA12A        202 T   VPAIWKQPAKQ   FMR   QAAyQ 3  AG   L                       224


HSPA9B         230 YGLDK s   edkv   2                                           239
HSPA8-1        183 YGLDK k 3 vgaer                                              193
HSPA5          209 YGLDK r   egek                                               218
HSPA4L         181 YGIYK q   dlppld   ek   pr                                   196
HYOU1-1        213 YGVFR r   kdintt   aq 3                                      226
HSPA14-a       180 YGIGQ d   sptgks 2                                           191
STCH           211 YGLHK a   dvf                                                219
HSPA12A        225       a   spense   ql   iialepeaasiycrklrlhqmielsskaavngysg  268


HSPA9B         240                                                  IAVYD  244
HSPA8-1        194                                                 NVLIFD  199
HSPA5          219                                                 NILVFD  224
HSPA4L         197                                                 NVVFID  202
HYOU1-1        233                                                 NIMFYD  238
HSPA14-a       192                                                 NILVFK  197
STCH           220                                                 HVLVID  225
HSPA12A        269 sdtvgagftqa 1 kehirrnrqsrtflvenvigeiwseleeg 1 d KYVVVD  315
```

Figure S3 (*continued 2*)

```
HSPA9B         245 LG GGTFDISILEIQ  KGVF e                         VKST ng      269
HSPA8-1        200 LG GGTFDVSILTIE  DGIF e                         VKST ag      224
HSPA5          225 LG GGTFDVSLLTID  NGVF e                         VVAT ng      249
HSPA4L         203 MG HSAYQVLVCAFN  KGKL k 3                       VLAT tf      227
HYOU1-1        233 MGsGSTV CTIVTYQ       m   vktkea   gmqpqlq   irg      vg 2 f  266
HSPA14-a       198 LG GTSLSLSVMEVN  SGIY r                         VLST nt      222
STCH           226 LG GGTLDVSLLNKQ  GGMF l   tramsg 1                           250
HSPA12A        316 SG GGTVDLTVHQIR       l   peghlk   elykatg 1 gp              345


HSPA9B         270  DTFLGGEDFD       QALL RHIVKEFKR   E 3 tgv  DLTKDNMALQR  307
HSPA8-1        225  DTHLGGEDFD       NRMV NHFIAEFKR   K   hkk  DISENKRAVRR  262
HSPA5          250  DTHLGGEDFD       QRVM EHFIKLYKK   K   tgk  DVRKDNRAVQK  287
HSPA4L         228  DPYLGGRNFD       EALV DYFCDEFKT   K   yki  NVKENSRALLR  265
HYOU1-1        267  DRTLGGLEME lrlr  ERLA GLFNEQRKG   Q   rak  DVRENPRAMAK  308
HSPA14-a       223  DDNIGGAHFT       ETLA QYLASEFQR 2 S   fkh  DVRGNARAMMK  260
STCH           251  NNKLGGQDFN       QRLL QYLYKQIYQ   T   yg   FVPSRKEEIHR  287
HSPA12A        346  YGSL GVDYE fekll YKIFgEDFIEQFKI   K   rpaa              378


HSPA9B         308  VREAAE  KAKCELSSSVQ 3 td                           326
HSPA8-1        263  LRTACE  RAKRTLSSSTQ                          ASI e   283
HSPA5          288  LRREVE  KAKRALSSQHQ                          ARI e   308
HSPA4L         266  LYQECE  KLKKLMSANAS   dlpln                          287
HYOU1-1        309 lLREA N  RLKTVLSANAD   hmaq  3                        329
HSPA14-a       261  LTNSAE  VAKHSLSTLGS                          ANC f   281
STCH           288  LRQAVEmvKLNLTLHQSAQ   ls                             308
HSPA12A        379                        wvdl    miafesrkraaapdrtnp LNI t   404


HSPA9B         327 IN LpYLTMD ssgpkh                                   341
HSPA8-1        284 IDSL YEGID                                          292
HSPA5          309 IESF YEGED                                          317
HSPA4L         288 IECF MNDLD                                          296
HYOU1-1        330 IEGL MDDVD                                          338
HSPA14-a       282 PDSL YEGQD                                          290
STCH           309 V LLtVEEQD rkephssdtelpkdklssaddhrvn   sgfgrglsdkksge  356
HSPA12A        405            lpfsfidyykkfrghsvehalrksn 2 vdfvkwssqgmlr   442


HSPA9B         342      LNMKLTR   AQFEGI vt   DLIRRTIAPCQKA MQDAE 3 v     395
HSPA8-1        293      FYTSITR   ARFEEL na   DLFRGTLDPVEKA LRDAK   l     326
HSPA5          318      FSETLTR   AKFEEL nm 3 DLFRSTMKPVQKV LEDSD   l     351
HSPA4L         297      VSSKMNR 2 AQFEQL ca   SLLAR VEPPLKAvMEQAN 1 l     330
HYOU1-1        339      FKAKVTR   VEFEEL ca   DLFERVPGPVQQA LQSAE   m     372
HSPA14-a       291      FDCNVSR 2 ARFELL cs   PLFNKCLEAI RG LLDQN   gf    324
STCH           357 sqvl FETEISR   KLFDTL ne   DLFQKILVPIQQV LKEGH   l     394
HSPA12A        443 mspda                 mna                        lfkp  454
```

Figure S3 (continued 3)

```
HSPA9B         396                     SK   SD IGE   VILVGGMTRMPKV   QQTVQDL  402
HSPA8-1        327                     DK   SQ IHD   IVLVGGSTRIPKI   QKLLQDF  353
HSPA5          352                     KK   SD IDE   IVLVGGSTRIPKI   QQLVKEF  378
HSPA4L         331                     QR   ED ISS   IEIVGGATRIPAV k EQITK F  357
HYOU1-1        373                     SL 3 D eIEQ   VILVGGATRVPRV   QEVLLKA  399
HSPA14-a       325                     TA   DD INK 3 VVLCGGSSRIPKL   QQLIKDL  351
STCH           395                     EK   TE IDE   VVLVGGSTRIPRI   RQVIQEF  421
HSPA12A        455 tidsiiehlr 1 dlfqkp EV   ST VKF   LFLVGGFAEAPLL   QQAVQAA  497


HSPA9B         403 F   GR    APSKAVNP D EAVAIGAA   IQ   GGVL agd            431
HSPA8-1        354 F n GK    ELNKSINP D EAVAYGAA 1 VQ   AAIL sgdksen        387
HSPA5          379 F n GK    EPSRGINP D EAVAYGAA   VQ   AGVL sg             407
HSPA4L         358 F   LK    DISTTLNA D EAVARGCA   LQ 3 CAIL spafkvrefs     393
HYOU1-1        400 V   GK 2 eELGKNINA D EAAAMGAV   YQ   AAAL skafkvkpfv     436
HSPA14-a       352 F p AV    ELLNSIPP D EVIPIGAA   IE   AGIL igkenll        385
STCH           422 F   GK    DPNTSVDP D LAVVTGVA   IQ   AGID ggswplqvsalei  460
HSPA12A        498 F   GD    QC RIIIPqD                    vgltilkgavlfg  522


HSPA9B         432     VTD        VLLLDVT                                     441
HSPA8-1        388     VQD        LLLLDVT                                     397
HSPA5          408     DQD tg 1 d LVLLDVC                                     420
HSPA4L         394     ITD lv  py                                             400
HYOU1-1        437     VRD av  vy         pil 3 veftrev   eeepgihslkhnkrvlf  470
HSPA14-a       386     VED sl  mi         ecs   ardilvk 3                     402
STCH           461 pnkh LQK tn  fn                                             471
HSPA12A        523 ldpa    vi  kv         rrs                                 533


HSPA9B         442
HSPA8-1        398
HSPA5          421
HSPA4L         401
HYOU1-1        471 srmgpypqrkvitfnryshdfnfhinygdlgflgpedlr 2 vfgsqnlttv  519
HSPA14-a       403
STCH
HSPA12A        534


HSPA9B         442
HSPA8-1        398
HSPA5          421
HSPA4L         401
HYOU1-1        520 klkgvgdsfkkypdyeskgikahfnldesgvlsldr 3 vesvfetlvedsa  568
HSPA14-a       403
STCH
HSPA12A        534
```

Figure S3 (*continued 4*)

```
HSPA9B         442    PLSL                                               445
HSPA8-1        398    PLSL                                               401
HSPA5          421    PLTL                                               424
HSPA4L         401    SITL rw   kt                                       408
HYOU1-1        569 ee ESTL tk 1 lgnt                                     580
HSPA14-a       403
STCH
HSPA12A        534    PLTY gv   gvlnryvegkhppekllvkdgtrwctdvfdkfisadqsva  579


HSPA9B         446                                   GIETLGG   VF  454
HSPA8-1        402                                   GIETAGG   VM  410
HSPA5          425                                   GIETVGG   VM  433
HSPA4L         409                                   SFEDGSG 2  E  416
HYOU1-1        581                                              I  581
HSPA14-a       403                                   GVDESGA sr F  412
STCH
HSPA12A        580 lgelvkrsytpakpsqlviviniyssehdnvsfitdp GVKKCGT lr    625


HSPA9B         455 TKLINRNTTIPTKKSQ 3 vfs   TAADGQTQVEIK   VCQG   EREMAGDNK  497
HSPA8-1        411 TVLIKRNTTIPTKQTQ   tft   TYSDNQPGVLIQ 3 VYEG   ERAMTKDNN  454
HSPA5          434 TKLIPRNTVVPTKKSQ   ifs   TASDNQPTVTIK   VYEG 1 ERPLTKDNH  477
HSPA4L         417 CEVFCKNHPAPFSKVI   tfhkk                                   437
HYOU1-1        582 SSLFGGGTT PDAKEN   gtdtv                                   601
HSPA14-a       413 TVLFPSGTPLPARR Q   htl   QAPGSISSVCLE   LYES d GKNSAKEET  456
STCH
HSPA12A        626


HSPA9B         499 LLGQ          FTL 3 IGIP         PAPRGVPQIEVTFDIDA   NGIVHVSAKD  536
HSPA8-1        455 LLGK          FEL   TGIP         PAPRGVPQIEVTFDIDA   NGILNVSAVD  492
HSPA5          478 LLGT          FDL   TGIP         PAPRGVPQIEVTFEIDV   NGILRVTAED  515
HSPA4L         438        epf               eleafy                                  446
HYOU1-1        602        q   3             eeeespa                                 609
HSPA14-a       457 KFAQ 3 vvl    QDL        d       KKENGLRDILAVLTMKR 2 DGSLHVTCTD  494
STCH
HSPA12A        626               LDL   TG   tsgtav  PARREI QTLMQFG DT   E I KATAID  659


HSPA9B         537 KG                                                        538
HSPA8-1        493 KS                                                        494
HSPA5          516 KG                                                        517
HSPA4L         447    tnlhevpypdarig 1 sftiqnvfpqsdgdsskvkvkvrvnihgifsv  492
HYOU1-1        610    egskdepgeqvelk  eeaeapvedgsqppppepkgdatpegekateke  656
HSPA14-a       495 QE                                                        496
STCH
HSPA12A        660 IA                                                        661
```

Figure S3 (*continued 5*)

```
HSPA9B         539
HSPA8-1        495
HSPA5          518
HSPA4L         493 asasviek   qnlegdhsdapmetetsfknenkdnm 3 dkmqvdqeeghqkch 541
HYOU1-1        657 ngdkseaq 3 kpsekaeagpegvapapegekkqkpa  rkrrm            695
HSPA14-a       497
STCH
HSPA12A        662


HSPA9B         539             TGREQQI 1  V  IQSSG   g                        552
HSPA8-1        495             TGKENKI   TI  TNDKG 1 r                        509
HSPA5          518             TGNKNKI   TI  TNDQN   r                        532
HSPA4L         542 aehtpeeeidh TGAKTK  3 SA  VSDKQ   drlnqtlkkgk VKSIDLPI     584
HYOU1-1        696                                               VEEIGVEL     703
HSPA14-a       497             TGKCEAI   SI          eias                     509
STCH
HSPA12A        662             TSKSVKV   GI          dflny                    675


HSPA9B         553         LSKDDIENMV kna      EKYAEEDRRKK 3 ERVEAVNMAEGIIH  590
HSPA8-1        510         LSKEDIERMV qea      EKYKAEDEKQR   DKVSSKNSLESYAF  547
HSPA5          533         LTPEEIERMV nda      EKFAEEDKKLK   ERIDTRNELESYAY  570
HSPA4L         585 qsslcrq LGQDLLNSYI ene 3    GKMIMQDKLEK   ERNDAKNAVEEYVY  629
HYOU1-1        704 vvldlpd LPEDKLAQSV  qk 2 l QDLTLRDLEKQ   EREKAANSLEAFIF  748
HSPA14-a
STCH
HSPA12A


HSPA9B         591 DTE   TKMEE fkdqlpa   DE 3 CNKLKEE  ISK mr ELLARK    DSE t  629
HSPA8-1        548 NMK   ATVED eklqgkin  DE   DKQKILDK CNE ii NWLDKNQ 3 TAE k  589
HSPA5          571 SLK   NQIGD keklggkls SE   DKETM EKaVEE ki EWLESH    QDA d  612
HSPA4L         630 DFR   DRLGT vyekfitp   E 3 DLSKLSAV LED te NWLYEDG   EDQ p  670
HYOU1-1        749 ETQ 3 DKLYQ peyqevst  EE   QREEISGK LSA as TWLEDEG   VGA t  790
HSPA14-a
STCH
HSPA12A


HSPA9B         630 g                                                          630
HSPA8-1        590
HSPA5          613 i                                                          613
HSPA4L         671 kq   vyv DKLGELK 3 Kyg QPI qmkym EHEE r PKALN dlgkkiqlvm   713
HYOU1-1        791 tv 3 mlk EKLAELR   Klc QGL ffrve ERKK w PERLS aldnllnhss   833
HSPA14-a
STCH
HSPA12A
```

Figure S3 (*continued 6*)

```
HSPA9B         631
HSPA8-1        590
HSPA5          614
HSPA4L         714 kvie   ayrnk 3 derydhldptemek  VEKCISDAM   SW lnskmnaqnkls  759
HYOU1-1        834 mflk 2 garli   pemdqiftevemtt  LEKVINETW 3 AW knatlaeqak    877
HSPA14-a
STCH
HSPA12A

HSPA9B         631              ENIRQA A   SSLQQASLKLFEMAY k     k 3        654
HSPA8-1        590              EEFEHQ Q   KELEKVCNPIITKLY q SAG g   mpggm  621
HSPA5          614              EDFKAK K   KELEEIVQPIISKLY g SAG p          640
HSPA4L         760 L TQD pvvkvs E IVAKsK 3  ELDNFCNPIIYKPK p     k          792
HYOU1-1        878 LpATE kpvlls KDIEAK M   MALDREVQYLLNKAK f     t          912
HSPA14-a
STCH
HSPA12A

HSPA9B         655 masere                                         660
HSPA8-1        622 pggfpgggappsggassgptieevd                      646
HSPA5          641 pptgeed                                        647
HSPA4L         793 aev                     PEDKPK AN S ehng PMD   811
HYOU1-1        913 k                       PRPRPKdKNgT raep PLN   931
HSPA14-a
STCH
HSPA12A

HSPA9B         661        gsgssgtge   qkedqkeekq                           679
HSPA8-1
HSPA5          648        taekdel                                          654
HSPA4L         812 GQSGTE tksdstkds   sqhtkssgemevd                        839
HYOU1-1        932 ASASDQ gekvippag 1 qtedaepisepekvetg 1 sepgdteplelggpg  978
HSPA14-a
STCH
HSPA12A

HSPA9B
HSPA8-1
HSPA5
HSPA4L
HYOU1-1        979 ae 1 peqkeqstgqkrplkndel  999
HSPA14-a
STCH
HSPA12A
```

Figure S4

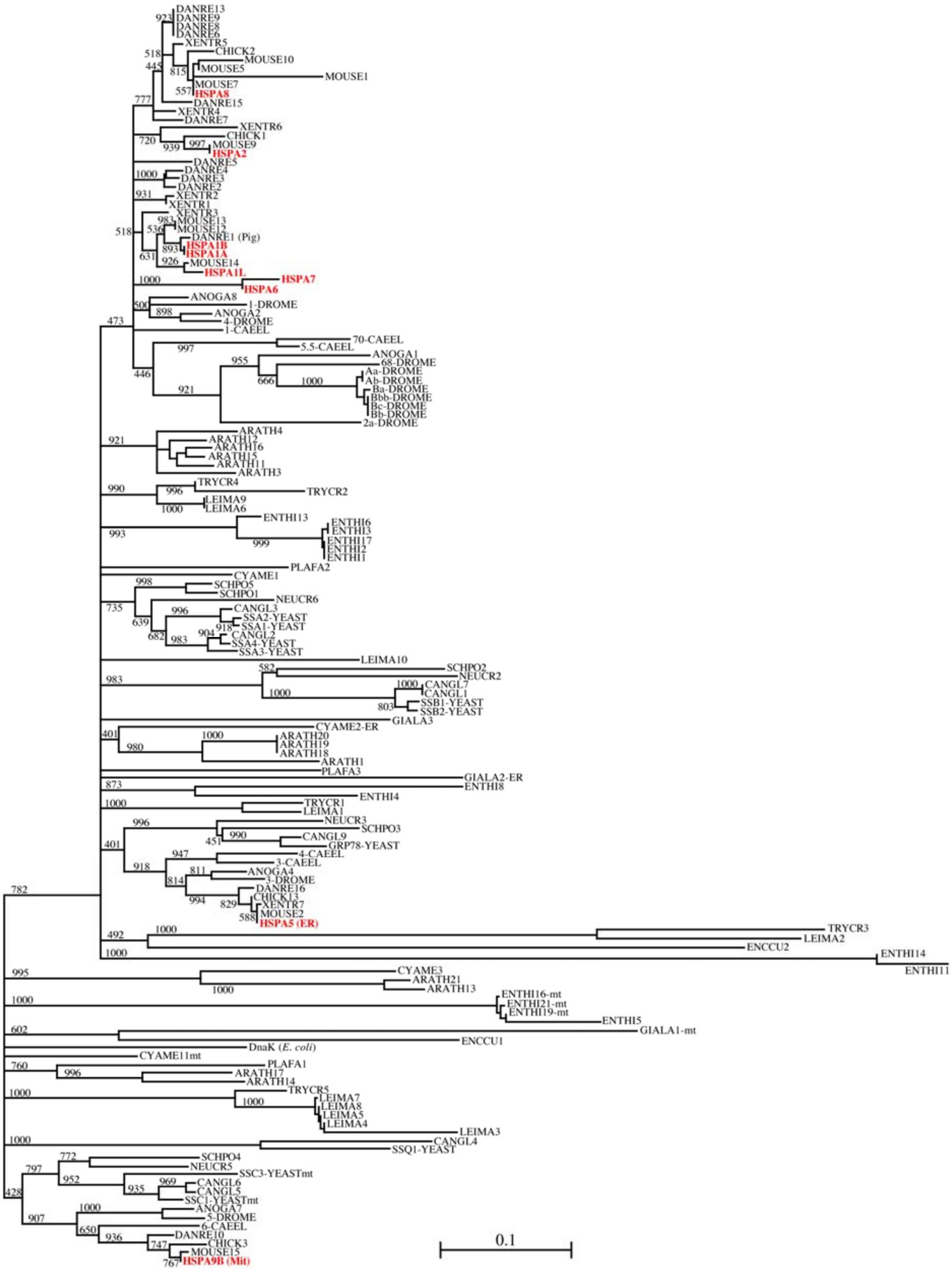

DANRE13
DANRE9
DANRE8
DANRE6
XENTR5
CHICK2
MOUSE10
MOUSE5
MOUSE1
MOUSE7
HSPA8
DANRE15
XENTR4
DANRE7
XENTR6
CHICK1
MOUSE9
HSPA2
DANRE5
DANRE4
DANRE3
DANRE2
XENTR2
XENTR1
XENTR3
MOUSE13
MOUSE12
DANRE1 (Pig)
HSPA1B
HSPA1A
MOUSE14
HSPA1L
HSPA7
HSPA6
ANOGA8
1-DROME
ANOGA2
4-DROME
1-CAEEL
70-CAEEL
5.5-CAEEL
ANOGA1
68-DROME
Aa-DROME
Ab-DROME
Ba-DROME
Bbb-DROME
Bc-DROME
Bb-DROME
2a-DROME
ARATH4
ARATH12
ARATH16
ARATH15
ARATH11
ARATH3
TRYCR4
TRYCR2
LEIMA9
LEIMA6
ENTHI13
ENTHI6
ENTHI3
ENTHI17
ENTHI2
ENTHI1
PLAFA2
CYAME1
SCHPO5
SCHPO1
NEUCR6
CANGL3
SSA2-YEAST
SSA1-YEAST
CANGL2
SSA4-YEAST
SSA3-YEAST
LEIMA10
SCHPO2
NEUCR2
CANGL7
CANGL1
SSB1-YEAST
SSB2-YEAST
GIALA3
CYAME2-ER
ARATH20
ARATH19
ARATH18
ARATH1
PLAFA3
GIALA2-ER
ENTHI8
ENTHI4
TRYCR1
LEIMA1
NEUCR3
SCHPO3
CANGL9
GRP78-YEAST
4-CAEEL
3-CAEEL
ANOGA4
3-DROME
DANRE16
CHICK13
XENTR7
MOUSE2
HSPA5 (ER)
TRYCR3
LEIMA2
ENCCU2
ENTHI14
ENTHI11
CYAME3
ARATH21
ARATH13
ENTHI16-mt
ENTHI21-mt
ENTHI19-mt
ENTHI5
GIALA1-mt
ENCCU1
DnaK (*E. coli*)
CYAME11mt
PLAFA1
ARATH17
ARATH14
TRYCR5
LEIMA7
LEIMA8
LEIMA5
LEIMA4
LEIMA3
CANGL4
SSQ1-YEAST
SCHPO4
NEUCR5
SSC3-YEASTmt
CANGL6
CANGL5
SSC1-YEASTmt
ANOGA7
5-DROME
6-CAEEL
DANRE10
CHICK3
MOUSE15
HSPA9B (Mit)
0.1

Table S1. Exon structure of transcripts from human *hsp70* genes

Name[a]	Group[b]	Location	S[c]	Exon Start / End[d]	Length/nt[e]	Splice site[f]
HSPA12A	I	10q25.3	-	118,491,931 / 118,492,075	145 (40)	14-1
				118,456,701 / 118,456,786	86	42-3
				118,454,652 / 118,454,779	128	85-2
				118,450,444 / 118,450,630	187	147-3
				118,448,136 / 118,448,240	105	182-3
				118,441,852 / 118,441,968	117	221-3
				118,433,292 / 118,433,463	172	279-1
				118,431,292 / 118,431,378	87	308-1
				118,430,653 / 118,430,757	105	343-1
				118,429,004 / 118,429,262	259	429-2
				118,425,900 / 118,426,003	104	464-1
				118,420,693 / 118,424,919	4227 (638)	
HSPA12B	I	20p13	+	3,661,356 / 3,661,441	*86*	
				3,667,305 / 3,667,364	60 (43)	15-1
				3,669,462 / 3,669,559	98	47-3
				3,670,931 / 3,671,055	125	89-2
				3,673,549 / 3,673,735	187	151-3
				3,674,140 / 3,674,244	105	186-3
				3,674,562 / 3,674,678	117	225-3
				3,676,864 / 3,677,038	175	284-1
				3,677,879 / 3,677,965	87	313-1
				3,678,397 / 3,678,501	105	348-1
				3,678,616 / 3,678,874	259	434-2
				3,679,451 / 3,679,554	104	469-1
				3,680,158 / 3,681,758	1601 (656)	
HSPA9B	II	5q31.2	-	137,938,826 / 137,939,014	189 (81)	27-3
				137,937,656 / 137,937,714	59	47-2
				137,937,351 / 137,937,438	88	76-3
				137,934,548 / 137,934,729	182	137-2
				137,932,513 / 137,932,637	125	179-1
				137,931,237 / 137,931,310	74	203-3
				137,931,043 / 137,931,149	107	239-2
				137,930,589 / 137,930,751	163	293-3
				137,930,214 / 137,930,306	93	324-3
				137,925,168 / 137,925,377	210	394-3
				137,923,452 / 137,923,679	228	470-3
				137,922,141 / 137,922,245	105	505-3
				137,921,457 / 137,921,574	118	545-1
				137,920,990 / 137,921,084	95	576-3
				137,920,362 / 137,920,454	93	607-3
				137,920,040 / 137,920,180	141	654-3
				137,918,923 / 137,919,702	780 (75)	

Table S1 (*continued 1*)

Name[a]	Group[b]	Location	S[c]	Exon Start / End[d]	Length/nt[e]	Splice site[f]
HSPA4-a	III	5q31.1	+	132,415,561 / 132,415,948	388 (107)	36-2
				132,428,571 / 132,428,628	58	55-3
				132,431,008 / 132,431,148	141	102-3
				132,433,965 / 132,434,087	123	143-3
				132,436,821 / 132,436,920	100	177-1
				132,437,584 / 132,437,717	134	221-3
				132,440,245 / 132,440,489	245	303-2
				132,450,373 / 132,450,449	77	329-1
				132,451,995 / 132,452,146	152	379-3
				132,452,646 / 132,452,752	107	415-2
				132,453,153 / 132,453,286	134	460-1
				132,454,784 / 132,454,965	182	520-3
				132,456,291 / 132,456,380	90	550-3
				132,459,689 / 132,459,841	153	601-3
				132,460,752 / 132,460,877	126	643-3
				132,463,136 / 132,463,243	108	679-3
				132,465,350 / 132,465,469	120	719-3
				132,467,456 / 132,467,617	162	773-3
				132,467,824 / 132,468,608	785 (204)	
HSPA4-b	III	5q31.1	+	132,415,561 / 132,415,948	388 (107)	36-2
				132,428,571 / 132,428,628	58	55-3
				132,431,008 / 132,431,148	141	102-3
				132,433,965 / 132,433,983	19	109-1
				132,467,906 / 132,468,608	703 (119)	
HSPA4L	III	4q28.1	+	128,922,903 / 128,923,262	360 (107)	36-2
				128,934,682 / 128,934,739	58	55-3
				128,936,373 / 128,936,513	141	102-3
				128,939,192 / 128,939,314	123	143-3
				128,941,743 / 128,941,842	100	177-1
				128,942,390 / 128,942,523	134	221-3
				128,944,234 / 128,944,478	245	303-2
				128,944,616 / 128,944,692	77	329-1
				128,945,678 / 128,945,829	152	379-3
				128,946,292 / 128,946,398	107	415-2
				128,948,601 / 128,948,734	134	460-1
				128,952,042 / 128,952,241	200	526-3
				128,958,930 / 128,959,025	96	558-3
				128,961,033 / 128,961,170	138	604-3
				128,963,374 / 128,963,499	126	646-3
				128,964,122 / 128,964,229	108	682-3
				128,967,910 / 128,968,029	120	722-3
				128,971,243 / 128,971,404	162	776-3
				128,973,288 / 128,973,972	685 (189)	

Table S1 (continued 2)

Name[a]	Group[b]	Location	S[c]	Exon Start / End[d]	Length/nt[e]	Splice site[f]
HSPH1-1,2	III	13q12.3	-	30,633,613 / 30,634,117	505 (107)	36-2
				30,630,943 / 30,631,000	58	55-3
				30,627,651 / 30,627,791	141	102-3
				30,626,770 / 30,626,892	123	143-3
				30,624,989 / 30,625,088	100	177-1
				30,623,746 / 30,623,879	134	221-3
				30,623,084 / 30,623,328	245	303-2
				30,622,091 / 30,622,319	229	379-3
				30,620,511 / 30,620,617	107	415-2
				30,620,096 / 30,620,229	134	460-1
				30,617,700 / 30,617,905	206	528-3
				30,615,929 / 30,616,060[g]	132	572-3
				30,613,259 / 30,613,396	138	618-3
				30,612,321 / 30,612,446	126	660-3
				30,611,137 / 30,611,244	108	696-3
				30,610,918 / 30,611,037	120	736-3
				30,610,544 / 30,610,705	162	790-3
				30,608,762 / 30,609,661	900 (207)	
HYOU1-1,2,3,4	III	11q23.3	-	118,433,074 / 118,433,122	*49* iso-1	
				118,432,941 / 118,433,035	*95* iso-2	
				118,431,992 / 118,432,089	98 (91) iso-1,2,4	31-1
				118,431,992 / 118,432,088	*97* iso-3	
				118,431,672 / 118,431,765	94 (80, iso-3)	62-2 / 27-2[h]
				118,431,415 / 118,431,493	79	88-3 / 53-3
				118,431,107 / 118,431,261	155	140-2 / 105-2
				118,430,906 / 118,430,982	77	166-1 / 133-1
				118,430,416 / 118,430,597	182	226-3 / 191-3
				118,430,043 / 118,430,158	116	265-2 / 230-2
				118,428,559 / 118,428,751	193	329-3 / 294-3
				118,428,217 / 118,428,351	135	374-3 / 339-3
				118,428,012 / 118,428,094	83	402-2 / 367-2
				118,427,741 / 118,427,874	134	446-3 / 411-3
				118,427,360 / 118,427,547	188	509-2 / 474-2
				118,426,957 / 118,427,095	139	555-3 / 520-3
				118,425,699 / 118,425,759	61	576-1 / 541-1
				118,425,478 / 118,425,557	80	602-3 / 567-3
				118,424,940 / 118,425,126[i]	187	664-3 / 629-3
				118,424,636 / 118,424,808	173	722-2 / 687-2
				118,424,398 / 118,424,485	88	751-3 / 716-3
				118,424,170 / 118,424,292	123	792-3 / 757-3
				118,423,869 / 118,424,002	134	837-2 / 802-2
				118,423,656 / 118,423,740	85	865-3 / 830-3
				118,422,484 / 118,422,724	241	946-1 / 911-1
				118,422,339 / 118,422,389	51	963-1 / 928-1
				118,421,703 / 118,421,753	51	980-1 / 945-1
				118,420,110 / 118,421,576	1,467 (62)	

Table S1 (continued 3)

Name[a]	Group[b]	Location	S[c]	Exon Start / End[d]	Length/nt[e]	Splice site[f]
HSPA14-a	IV	10p13	+	14,920,267 / 14,920,464	198 (57)	19-3
				14,921,910 / 14,921,990	81	46-3
				14,922,080 / 14,922,162	83	74-2
				14,930,614 / 14,930,662	49	90-3
				14,930,795 / 14,930,900	106	126-1
				14,931,726 / 14,931,816	91	156-2
				14,933,224 / 14,933,328	105	191-2
				14,934,375 / 14,934,536	162	245-2
				14,936,130 / 14,936,285	156	297-2
				14,937,846 / 14,937,948	103	331-3
				14,949,088 / 14,949,300	213	402-3
				14,949,741 / 14,949,914	174	460-3
				14,952,602 / 14,952,672	71	484-2
				14,953,533 / 14,953,746	214 (79)	
HSPA14-b	IV	10p13	+	14,920,334 / 14,920,464	131 (57)	19-3
				14,921,910 / 14,921,990	81	46-3
				14,922,080 / 14,922,162	83	74-2
				14,924,139 / 14,924,851	713 (46)	
				14,925,376 / 14,926,753	*1378*	
STCH	V	21q11.2	-	14,677,287 / 14,677,380	94 (25)	9-1
				14,675,395 / 14,675,735	341	122-3
				14,672,391 / 14,672,604	214	194-1
				14,669,844 / 14,670,011	168	250-1
				14,665,307 / 14,668,476	3170 (668)	
HSPA8-1	VI	11q24.1	-	122,437,982 / 122,438,054	*73*	
				122,437,038 / 122,437,247	210 (205)	69-1
				122,436,511 / 122,436,716	206	137-3
				122,436,034 / 122,436,186	153	188-3
				122,435,391 / 122,435,946	556	374-1
				122,434,977 / 122,435,179	203	441-3
				122,434,550 / 122,434,748	199	508-1
				122,434,170 / 122,434,402	233	585-3
				122,433,410 / 122,433,837	428 (186)	
HSPA8-2	VI	11q24.1	-	122,437,982 / 122,438,054	*73*	
				122,437,038 / 122,437,247	210 (205)	69-1
				122,436,511 / 122,436,716	206	137-3
				122,436,034 / 122,436,186	153	188-3
				122,435,391 / 122,435,946	556	374-1
				122,434,977 / 122,435,179	203	441-3
				122,434,685 / 122,434,748	64	463-1
				122,433,410 / 122,433,746	337 (95)	

Table S1 (continued 4)

Name[a]	Group[b]	Location	S[c]	Exon Start / End[d]	Length/nt[e]	Splice site[f]
HSPA2	VI	14q23.3	-	64,072,376 / 64,072,446<br>64,077,316 / 64,079,708	*71*<br>2393 (1920)	
HSPA1A	VI	6p21.33	+	31,891,316 / 31,893,698	2,383 (1,926)	
HSPA1B	VI	6p21.32	+	31,903,503 / 31,906,010	2,508 (1,926)	
HSPA1L	VI	6p21.33	-	31,890,645 / 31,890,814<br>31,885,375 / 31,887,741	*170*<br>2,367 (1,926)	
HSPA6	VI	1q23.3	+	159,760,660 / 159,763,311	2,652 (1932)	
HSPA7[j]	VI	1q23.3	+	?<159,842,705 /<br>159,843,718 + 159,843,720<br>/ 159,844,631>?	1014 +<br>912 (1,927)	
HSPA5	VII	9q33.3	-	127,043,105 / 127,043,430<br>127,042,776 / 127,043,007<br>127,042,272 / 127,042,409<br>127,041,521 / 127,041,633<br>127,041,041 / 127,041,431<br>127,040,690 / 127,040,927<br>127,040,241 / 127,040,408<br>127,036,953 / 127,039,254	326 (122)<br>232<br>138<br>113<br>391<br>238<br>168<br>2302 (563)	41-2<br>118-3<br>164-3<br>202-2<br>332-3<br>412-1<br>468-1

[a]Gene name as in NCBI Entrez Gene database with alternatively spliced/transcribed variants hyphenated at the end of the name.

[b]Group according to our classification.

[c]DNA strand.

[d]Positions encoding the exon along the genome sequenced strand. These correspond to the 5’ / 3’ termini for exons encoded on the direct strand, and to the 3’ / 5’ termini for exons encoded on the complementary strand.

[e]Length in nucleotides of the exon. In parenthesis is the length of the coding part in the first and last coding exon. Non-coding exons are in italics.

[f]Position of the splice site within the coding sequence. Position *k*-*i* means that the splice site is after the *i*-th nucleotide (*i*=1,2,3) of codon *k*.

[g]Exon XII skipped in HSPH1 isoform b.

[h]First set of codon positions refers to isoforms 1 and 2 of HYOU1. Second set of positions refers to isoform 3.

[i]Last exon match for HYOU1-4 (deduced from protein Q6IN67_HUMAN). No genomic match found for the last 14 C-terminal residues.

[j]Sequence encoded by two segments with one frame-shift.
